# Identification of Fish Species and Targeted Genetic Modifications Based on DNA Analysis: State of the Art

**DOI:** 10.3390/foods12010228

**Published:** 2023-01-03

**Authors:** Eliska Cermakova, Simona Lencova, Subham Mukherjee, Petra Horka, Simon Vobruba, Katerina Demnerova, Kamila Zdenkova

**Affiliations:** 1 Department of Biochemistry and Microbiology, University of Chemistry and Technology, 166 28 Prague, Czech Republic; 2 Department of Chemistry, Biochemistry and Food Microbiology, Food Research Institute Prague, 102 00 Prague, Czech Republic; 3 Institute for Environmental Studies, Faculty of Science, Charles University, 128 00 Prague, Czech Republic

**Keywords:** DNA-based methods, fish, food fraud, food quality, genetically modified organism (GMO), polymerase chain reaction (PCR), species identification

## Abstract

Food adulteration is one of the most serious problems regarding food safety and quality worldwide. Besides misleading consumers, it poses a considerable health risk associated with the potential non-labeled allergen content. Fish and fish products are one of the most expensive and widely traded commodities, which predisposes them to being adulterated. Among all fraud types, replacing high-quality or rare fish with a less valuable species predominates. Because fish differ in their allergen content, specifically the main one, parvalbumin, their replacement can endanger consumers. This underlines the need for reliable, robust control systems for fish species identification. Various methods may be used for the aforementioned purpose. DNA-based methods are favored due to the characteristics of the target molecule, DNA, which is heat resistant, and the fact that through its sequencing, several other traits, including the recognition of genetic modifications, can be determined. Thus, they are considered to be powerful tools for identifying cases of food fraud. In this review, the major DNA-based methods applicable for fish meat and product authentication and their commercial applications are discussed, the possibilities of detecting genetic modifications in fish are evaluated, and future trends are highlighted, emphasizing the need for comprehensive and regularly updated online database resources.

## 1. Introduction

Food adulteration, the act of misleading customers for financial gain, is currently one of the most discussed topics in the field of food analysis. It is a substantial concern that poses significant risks to public health and reduces food quality and nutrition value [1]. Fish and fish products are among the most commonly adulterated foods, which is driven by their increasing consumption worldwide, and fish population decline due to overfishing, along with the subsequent commercial consequences. Additionally, fish are among the most easily adulterated foods because of the morphological changes that occur during processing, which make visual identification impossible [2,3,4,5].

Fish and fishery products are a highly valuable source of nutrients. Their muscle is rich in both macro- and micro-nutrients, especially (i) proteins, (ii) vitamins, (iii) polyunsaturated omega-3 and omega-6 fatty acids important for the human neural system, and (iv) minerals, such as calcium, iodine, zinc, iron, and selenium [6]. Simultaneously, fish meat is usually low in saturated fats, carbohydrates, and cholesterol, and low in purines compared to other types of meat. This all makes fish an irreplaceable dietary component.

Despite all of the benefits of fish consumption, it is necessary to mention some of its risks. In addition to the concern about eating small bones in meat, there is the major issue of food allergy. Fish-induced allergy is a severe worldwide problem, as evidenced by an estimated prevalence of 7% in the pediatric population, often persisting into adulthood with serious symptoms [7,8]. Fish allergy is a pathophysiological immune response to specific proteins mediated by IgE, commonly manifesting as oral allergy syndrome, diarrhea, abdominal pain, rhinitis, angioedema, and several other symptoms, even as life-threatening anaphylaxis [9]. Allergenicity differs across fish species due to the content of specific proteins (main allergens) [10,11]. Parvalbumins are the leading ones; enolases, aldolases and gelatin have also been identified as fish allergens, although their role in fish allergenicity is still not clearly understood [9,12]. 

Parvalbumins are highly stable proteins with a low molecular weight (10–12 kDa) common in fish muscle [13]. Fish contains both α and β parvalbumins, with most allergens belonging to the β line [14]. The expression of β parvalbumins occurs mainly in the sarcoplasmic part of fish white muscle tissue. This is directly related to the allergenicity of individual species; for example, carp, which is mainly composed of white tissue, contains an up to 100 times higher level of the parvalbumins than tuna (Thunnus) or mackerel (Scomber), which primarily contain red tissue and therefore have a lower allergenicity potential [9]. Furthermore, there are various known paralogs of parvalbumins which cause allergies differently and play a physiological role in fish muscle adaptation to environmental influences. For example, salmon (Salmo) contains only one allergenic parvalbumin isoform, parvalbumin β1 [15]. In addition, food processing may influence fish allergenicity by parvalbumin degradation or oligomerization, which may change the IgE epitope number [9,16,17]. Generally, two conclusions can be drawn on the allergenicity of parvalbumins based on current knowledge: (i) parvalbumins from different species can vary in allergenicity; and (ii) parvalbumin isoforms from the same species can vary [9]. This all encourages consumer demand for a verification of food identity, and food inspection authorities to implement reliable, strict quality control mechanisms [2]. 

Various methods, differing in their principle and detected target molecules, can be used for fish species identification and adulteration detection. Recently, parvalbumin detection has been preferred, but this is quite demanding compared to other food allergens because of its high biochemical and immunological variability among fish species and differing thermostability. This in itself sidelines the use of some types of methods, e.g., proteomic ones, and favors DNA-based methods, whose target, DNA, is not destroyed by the processing. Using DNA analysis, for example, a common form of adulteration—replacing the species (a more expensive one with a cheaper one)—can be detected. Additionally, DNA-based methods have the potential to detect the consumer being misled about the fish’s origin, another common type of adulteration. This is even more relevant, since genetically modified (GM) fish can be bought on the world market. Even though GM organisms do not pose a threat to public health and their quality is no different, consumers should be informed about the content of GM products, and because the public can be skeptical about GM products, sellers may want to hide this information and deliberately deceive the consumer.

For the aforementioned reasons, it is essential to have reliable methods for fish species identification and the detection of adulteration. In this comprehensive review, the potential of DNA-based methods is critically evaluated, and all of the crucial aspects are discussed. The scope ranges from a summary and specification of DNA-based methods, through their commercial applications, to highlighting the need for further innovations and outlining the future challenges. In addition, current methodologies for the detection of GM organisms (GMOs) are examined.

## 2. Identification of Fish Species

Traditional fish species identification has been based on the qualitative and quantitative analysis of morphological features. Qualitative evaluation is usually focused on elementary characteristics such as fish body shape, fin placement, color, or the position of the mouth and possibly whiskers; quantitative morphological features commonly include the length of various body parts, number of scales, number of vertebrae or bones, etc. All of the above-mentioned characteristics are specific for a certain group of fish, depending on the nature of the species’ environment and the trophic resource use of the species (especially on the method it uses to obtain food). For this reason, it is still one of the methods used for the fast determination of fish species. However, the application of these attributes is not possible for the majority of processed products, in which fish meat is usually mixed with other ingredients and the morphological characteristics cannot be evaluated. Additionally, it is not possible to determine the areas and/or geographical origin of fish in processed products. This all emphasizes the need for the development of reliable analytical methods that enable fish species identification and that can detect food adulteration and consumers being misled. 

In general, various methods for the detection of fish adulteration have been developed over the last few decades. These primarily include gas and liquid chromatography [18,19,20], mass spectrometry [21,22], infrared spectroscopy [23], nuclear magnetic resonance (NMR) [24,25], immunochemical methods (e.g., Enzyme-Linked Immuno Sorbent Assay—ELISA) [26], and electrophoresis [18,27]. Each of them is based on a different principle and thus provides certain possibilities for detecting different means of food adulteration. For example, to determine a fish origin region, stable isotopes of calcified structures can be analyzed [28]. Furthermore, the analysis of δ13C isotopes by NMR spectroscopy allows the differentiation between wild and farmed fish [24]. For instance, organically farmed and conventionally farmed salmonid fish can be distinguished by the isotopes δ15N and δ13C [29]. However, species substitution is one of the most common types of fish adulteration, and thus analytical methods enabling fish species identification are of critical importance.

Currently, fish species identification is mostly performed by proteomic and/or genomic methods. Both approaches are reliable for the analysis of fresh or thawed tissue, but also have their specifications and limitations. Protein-based methods may fail to analyze heat-treated or dried samples due to the denaturation of proteins (occurring after the heating at 40–60 °C), causing the loss of their biological functions and changes in many of their properties. Physical and chemical conditions can also damage DNA molecules, but unlike proteins, DNA fragments contain sufficient differences in their sequence that allow species characterization [30,31]. Furthermore, the nucleotide sequence enables the identification of species from all cell types and is independent of the tissue source or sample damage. Other significant benefits of the DNA-based methods include their specificity, sensitivity, and speed [3,4,32]. This has all led to their frequent application and predominance over all of the above-mentioned methods [33,34,35].

## 3. DNA-Based Methods

**Figure 1:**
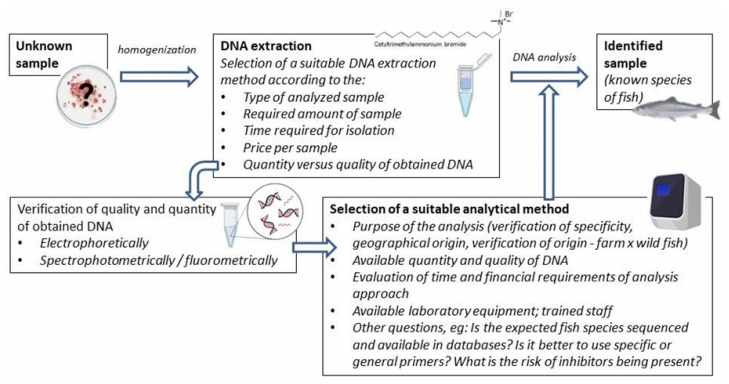
Scheme of the general procedure using DNA-based methods.

Various DNA-based methods are used for fish species identification. Although they are often based on different bases, their implementation includes several similar preparatory steps, such as DNA isolation and in silico analysis using available databases (e.g., designing specific primers). The general procedure is shown in Figure 1.

Of the DNA-based methods which have been developed so far, the most significant for fish species identification are the following (sorted alphabetically): Amplified Fragment Length Polymorphism (AFLP), DNA barcoding, Forensically Informative Nucleotide Sequencing (FINS), High-Resolution Melting (HRM) analysis, Polymerase Chain Reaction (PCR), Random Amplified Polymorphic DNA (RAPD), Restriction Fragment Length Polymorphism (RFLP), or Single-Stranded Conformational Polymorphism (SSCP); some of them are already available for commercial applications [3,36,37,38,39]. Over the last few decades, the Loop-Mediated Isothermal Amplification (LAMP) method, commonly used for the detection of a fish virus or seafood species [40,41], has been also used for identifying fish species [42,43,44,45]. Several methodologies have been carried out using either nuclear DNA (nDNA) or mitochondrial DNA (mtDNA). Thus, complex and regularly updated databases of genome sequences facilitating the selection of appropriate target molecules and identification markers are crucial for most of the methods. 

All of the above-mentioned aspects that are crucial for successful analysis are discussed below. The available databases are summarized and discussed in Section 3.1. The advantages and disadvantages of using both types of DNA—nDNA and mtDNA—are summarized in Section 3.2.; individual methods are discussed in more detail in Section 3.3.

### 3.1. Nucleic Acid Databases

The field of DNA sequencing technology has a rich history. In the past, DNA barcoding and FINS were widely employed, providing information mainly about mtDNA. This was followed by WGS, which together with mtDNA information was able to provide the complete information regarding the organisms studied. The overwhelming production of DNA sequences led to the necessary introduction of nucleic acid databases.

The Fish Barcode of Life Initiative (FISH-BOL), a global effort to coordinate and standardize a reference sequence library for fish species, aimed to create a reliable, fast and cost-effective way to identify fish species based on DNA analysis. For this purpose, a public database of standardized mtDNA reference sequences was created in 2005 [46]. Although it is not complete, and the number of sequenced fish species is constantly growing [37,47], the FISH-BOL website is no longer available today; the obtained sequenced data are available in the Barcode of Life database (BOLD).

Currently, there are several databases of nucleic acid sequences. The most important ones are (i) the European Molecular Biology Laboratory (EMBL), which was established in 1980 as the first nucleotide sequence database in the United Kingdom, and which to this day is publicly available at the website of the European Bioinformatics Institute (http://www.ebi.ac.uk, accessed on 20 October 2022); (ii) the DNA Data Bank of Japan (DDBJ), managed by the Center for Information Biology (CIB), which mainly collects data from Japanese research (publicly available at http://www.ddbj.nig.ac.jp, accessed on 20 October 2022); and (iii) the GenBank database, maintained by the National Center for Biotechnology Information (NCBI), available at http://www.ncbi.nlm.nih.gov, accessed on 20 October 2022.

There are also databases that focus directly on fish nucleotide sequences, such as the European Union database FishTrace (https://fishtrace.jrc.ec.europa.eu/, accessed on 20 October 2022), which is limited to European marine fish species. Unlike FISH-BOL, which focused on COI sequences only, FishTrace includes sequences for both mitochondrial (cyt*b*) and nuclear (rhodopsin) gene. All of the databases contain the same set of information, including species names, collection records or sample identifiers. However, they differ in the area of the genetic material, which can lead to complications when comparing information to identify fish species. There are also differences in the measures for ensuring the quality of records in the database. For example, GenBank and European Nucleotide Archive (www.ebi.ac.uk/ena, accessed on 20 October 2022) do not require confirmation of the identification of the species whose sequences is to be uploaded to the database, whereas BOLD requires curated sequences. Additionally, the FishTrace database associates all data with expert-verified material [48]. Furthermore, NCBI automatically retrieve data from other databases, whereas in GigaDB (http://gigadb.org/, accessed on 20 October 2022), all datasets and metadata are manually curated according to guidelines provided by the Genomics Standards Consortium (www.gensc.org, accessed on 20 October 2022).

Knowledge of a fish genome sequence can significantly facilitate progress in the development of DNA-based identification methods. The first sequenced fish genome was Fugu (*Takifugu rubripes*) in 2002 [49], and the number of newly sequenced species rose slowly over the next few years. A breakthrough came in 2020, when more than 300 fish genomes were published [50], and this trend has continued to this day. In April 2022, the whole genome sequence (WGS) was available for almost 900 fish species, mainly in the NCBI repository and to a lesser extent also in other repositories, e.g., GigaDB and the ENA. Even though this is an impressive number, it represents less than 3% of the 32,000 total fish species on record [51].

In this review, we retrieved genome attribute data for all of the sequenced members of 15 fish orders that contain commercially significant species (Supplementary Table S1) [52,53]. All of the chosen orders belong to the class of Actinopterygii (ray-finned fishes). The orders Cichliformes and Perciformes were the most abundant, with 570 and 128 species with published WGS. Nevertheless, other orders are arguably more important in terms of the commercial significance and food adulteration, especially in the European Union—large pelagic Salmoniformes (15 species with WGS), Scombriformes (seven species) and Istiophoriformes (two species), forage Clupeiformes (eight species), demersal Gadiformes (28 species), Anguilliformes (six species) and Pleuronectiformes (17 species) and mixed Cypriniformes (54 species). Other important orders are Anabantiformes (five species), Esociformes (six species), Gobiiformes (13 species), Siluriformes (17 species) and Spariformes (five species).

With the increasing availability of sequencing and the need for fish species identification, significantly more fish WGS are expected to be published in the coming years. Besides smaller projects, the Fish10K Genome Project was announced in 2020. This project aims to sample, sequence, assemble, and analyze the genomes of 10,000 representative fish species over the next 10 years [54].

Similar to the WGS, knowledge of the mtDNA sequence can also be used for the identification of fish species (see Section 3.2.). By April 2022, the mtDNA sequences of 3297 fish species had been published in the NCBI repository. Almost all species with known WGS have had their mtDNA sequenced, as well, with several exceptions belonging to the orders Gadiformes and especially Cichliformes. Nevertheless, the reason for this discrepancy in those two orders remains unknown.

### 3.2. Mitochondrial and Nuclear Identification Markers

Identification markers are defined as parts of DNA sequences unique to a given species. These markers must meet the basic requirements for analysis. First of all, they must have sufficient specificity for a particular species to be identified. The choice of a suitable marker for a given purpose depends on the requirements, in particular, whether a qualitative or quantitative analysis is being performed. Various mitochondrial and nuclear markers, with different advantages and disadvantages, are currently used for this purpose.

For the identification of fish species, mitochondrial loci have been preferred to nuclear genes because of their features: mitochondrial genes belong to a haploid genome, they are present in high copy numbers (particularly in fish muscle tissues), which provides higher sensitivity of detection, and their mutation rate is greater than that of nuclear genes [55,56,57]. This means that the coalescence of neutral genes will be positively correlated with the adequate population size of the species [58]. Thus, according to population genetics theory, mtDNA should evolve four times faster than the average nuclear gene. Hence, mtDNA can be used to follow divergence in very closely related taxa and even within species. An undeniable advantage of mtDNA is the ability to identify the geographical origin of the individual [59,60].

The most common mitochondrial markers used for fish species identification are the following: (i) the gene for cytochrome-c-oxidase subunit I (COI; EC 7.1.1.9), whose 600-bp-long segment became the basis for taxonomic fish differentiation via DNA barcoding [61,62], and (ii) cytochrome b (cytb; EC 7.1.1.8) [63,64,65]. Due to the presence of a high copy number of mtDNA molecules in the cell (~1000× more than copies of nDNA), their occurrence is also expected in highly processed products [56,66]. Furthermore, mtDNA is thought to be more thermally stable due to the greater internal stability caused by its ring structure [67]. However, significant limitations have been noted with these markers [66]. The disadvantage of mtDNA sequencing is the potential occurrence of nuclear mitochondrial pseudogenes (numts). Numts, non-functional nuclear sequences of mitochondrial origin, can be found in a variety of metazoans, among others in crustaceans [68,69,70]. The amplification of these nuclear sequences, instead of or in addition to the mitochondrial sequence, can lead to ambiguous sequences, incorrect phylogenetic replacements or misinterpretation as frameshift mutation. Numts can be detected by checking for the occurrence of these effects, thus preventing erroneous results [71,72].

Another disadvantage of mitochondrial markers in comparison with nuclear ones is the impossibility of quantifying DNA, because mtDNA concentrations vary depending on the type of tissue [60,73].

The above-mentioned difficulties can be overcome by amplifying nuclear sequences instead, as they have the advantage of possessing a known haploid genome size and quite a high level of uniqueness, even in orthologs of such markers in closely related fish species [55,74]. This phenomenon, based on an absence of selection pressure on introns as they are spliced out and are not reflected in the resulting protein, allows for a high level of mutations, and therefore early diversification of their sequence after the split into new species [75]. In addition, nuclear DNA is contained in each cell depending on the number of nuclei, which is almost always the same for a given species [73]. The identification of fish through nuclear marker analysis thus enables not only qualitative but also quantitative analysis. This fact can be used in the analysis of the fish content in processed products, which is very useful with today’s growing popularity of fish products [60].

As for nuclear markers, the most frequently used ones for fish authentication are β-actin, which has also become an often-used internal control for mRNA quantification [76,77,78], and parvalbumin [79]. Besides these markers, novel nuclear barcode regions have also been proposed for fish species identification [80]. The length of these nDNA barcodes is generally shorter than that of the mtDNA barcodes: this facilitates the amplification of the DNA even for highly processed food products, as well as their compatibility with the current next-generation sequencing (NGS) technologies, which also allow the identification of species in a mixture [56].

Another frequently used marker for species identification are microsatellites, also called STRs (short tandem repeats) [81] or SSR (simple sequence repeats) [82,83]. Microsatellites are short stretches of DNA composed of repeating specific motifs of nucleotide sequences. These motifs are 1–10 nucleotides long. The majority of microsatellites in the genome (30–60%) are probably dinucleotide repeats; the most common motifs in the vertebrate genome are (AC)n or (AT)n [83]. In trinucleotide microsatellites, the most common sequences are (AAT)n and (CAG)n [84]; the sequences (GATA)n and (GACA)n are almost exclusively in tetranucleotides [85]. The number of repetitions of a unit (repeat) at a particular DNA site (locus) defines an allele. The allele length can be determined by PCR amplification of a given locus using primers adjacent to the microsatellite sequence. It is therefore necessary to know the surrounding sequences for the analysis of microsatellites. The PCR fragments are then separated by length in an automated sequencer by so-called fragmentation analysis.

Microsatellites are considered to be one of the most suitable genetic markers due to their extreme variability (polymorphism), multiallelic nature and codominant inheritance (this makes it possible to distinguish heterozygotes). Another advantage is their abundance across the genome, which requires a small amount of DNA to collect data [82,86]. Nuclear microsatellites are often highly species specific; they are thus used for analyses at the intra-species level. Sometimes microsatellites also occur in mtDNA.

In population biology, it is used for the identification of related individuals, as well as for the derivation of demographic parameters.

A comprehensive overview of genetic markers used to identify fish species, including information on the detection method, is given in Table 1.

### 3.3. Overview of DNA-Based Methods

**Figure 2:**
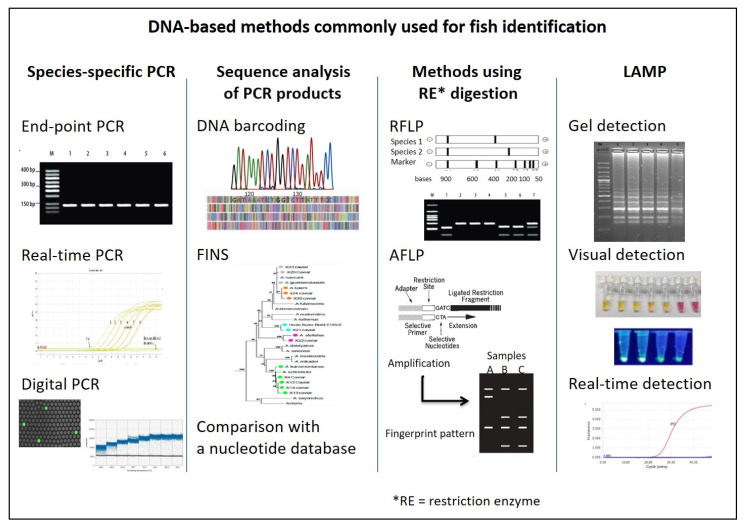
Schematic representation of commonly used DNA-based methods and data analysis approaches for fish species identification. PCR = Polymerase Chain Reaction; FINS = Forensically Informative Nucleotide Sequencing; RFLP = Restriction Fragment Length Polymorphism; AFLP = Amplified Fragment Length Polymorphism; LAMP = Loop-Mediated Isothermal Amplification.

DNA-based methods used for species identification are based on consistent genetic differences between species. The most prominent ones are those which use the polymerase chain reaction (PCR) principle to enrich the required DNA segment for analysis. However, various other methods can be used that have the potential to compete with PCR in the future in the best sense of the word. This chapter briefly summarizes the principles of selected methods and their advantages and/or disadvantages. The most commonly used methods for fish species identification are presented in Figure 2.

#### 3.3.1. Polymerase Chain Reaction (PCR)

The PCR method is used for the selective amplification of a short region of nucleic acid. It has become very popular and, therefore, has been constantly evolving and improving since its discovery in 1983 [148].

The method has gradually developed into several variants, namely end-point PCR, quantitative PCR with fluorescence detection in real time (qPCR), and digital PCR (dPCR) [149,150,151]. The basic principle of the method remains the same for all variants. In general, specific sequence amplification is based on in vitro enzymatic replication that is repeated cyclically. The amplified region (amplicon) is defined by two short oligonucleotides—PCR primers. Each primer binds to single strand of DNA and, subsequently, allows DNA polymerase to begin synthesizing a strand complementary to it. This creates a double-stranded DNA, which is separated into two single-stranded molecules by following denaturation at high temperature. The amplification process is exponential in nature. After the amplification, the detection of the PCR product depends on the type of PCR used.

The end-point PCR requires post-PCR electrophoretic detection on an agarose gel, which allows identification of amplicons by size. After qPCR, agarose electrophoresis is possible if needed, but not required. The qPCR, and also dPCR, allow the detection and quantification of target DNA in the reaction through the detection of fluorescence signals, which increases the sensitivity of the method. The fluorescence signal is produced by intercalating dye, such as SYBR Green I or Eva Green, or by a fluorescent dye-labeled probe.

The result of each qPCR is an amplification curve showing the increase in fluorescence, and thus the amount of product, in time. In the case of intercalating dye, the specificity can be lower because the dye binds to all double-strand products present in the reaction [150]. Therefore, post-PCR identification of fish species using High-Resolution Melting (HRM) analysis of the amplified gene segment (amplicons) is widely used [39,152,153]. HRM relies on the different melting temperatures (Tm) of amplicons that occur due to minor variations in nucleotide composition, especially on the number of guanine and cytosine, and the length of the sequences. The dsDNA melts as the temperature increases. Thus, the DNA-binding fluorescent intercalation dye is released, and the melting profiles can be recorded and systematically and statistically processed using specific HRM software. Based on the different Tm values and/or the shape of the curve, it is possible to distinguish between closely related species even when a single base variation is present between their dsDNA sequences [154,155,156]. HRM is an advanced method that provides a high level of confidence and accuracy, but it lacks the determination of the precise nucleotide differences in the analyzed amplicons [152,153,156,157,158]. This can be solved by sequencing the amplicons (see Section 3.3.2 and Section 3.4).

There is no melting curve analysis in digital PCR. When using an intercalating dye, the possible risk of non-specific results must be considered. However, a specific feature of dPCR is the division of the reaction mixture with the analyzed DNA into a large number of aliquots (drops or cells on the chip), in which the reaction itself takes place. End-point fluorescence is measured in each aliquot, which makes it possible to statistically evaluate the results achieved, and absolute quantification occurs [159,160].

In addition to sensitivity, specificity, and speed, the need for a small amount of template DNA or a small reaction volume are the general advantages of PCR. On the other hand, it is essential to have sufficient knowledge to design suitable primers and thus avoid the formation of non-specific products, primer dimers or hairpins [149,161,162]. Furthermore, one pair of species-specific primers is usually used for the identification of one species. Therefore, several primer pairs may be required.

Due to its properties and simple design, the PCR method is widely used for species identification. PCR is very effective in the analysis of mixed samples, even heat-treated ones. PCR also allows the detection of genetic modifications in selected genes (see Section 3.7). Nowadays, the qPCR method is most commonly used for these purposes. This is a cheap and sensitive method for differentiating similar species, and in addition, due to the precise determination of the number of target region copies in the reaction, it is easier to compare the quantitative results between laboratories and thus have better control over the analysis itself. The method is also used to multiply the target region, which is then sequenced (see Section 3.3.2) or digested by an enzyme (see Section 3.3.4). Comparing the obtained nucleotide sequences with the database makes it possible to identify the species from which the DNA originates. Moreover, the principle of PCR is the cornerstone of most other methods, such as sequencing or AFLP, and is thus an important method in general. Currently, PCR is considered a “gold standard” of DNA-based methods.

#### 3.3.2. Sequencing Methods

Today, many sequencing technologies exist. Traditional Sanger sequencing, which is limited by its ability to sequence only one DNA fragment at a time and lower sensitivity, has been overtaken by modern approaches using massive parallel sequencing based on NGS. NGS platforms can be roughly divided into long-reading (about 1 kb and above), such as MinION, and short-reading (usually <300 bases/read) sequencing platforms, e.g., Illumina, Ion Torrent, or Pyrosequencing, that provide higher throughput and are most suitable for PCR amplicon sequencing [163,164,165,166].

For fish species identification, Sanger sequencing and the Illumina platform are the most commonly used sequencing techniques. Both sequencing techniques, the Sanger dideoxy method (also known as capillary electrophoresis sequencing) and Illumina NGS, use the principle of amplification, in which DNA polymerase adds fluorescently labeled nucleotides to the growing strand one after the other, and the inserted nucleotide is subsequently identified due to its fluorescent label.

The advantage of Sanger sequencing is its speed and the ability to sequence relatively long fragments (up to 1000 bp). It is also cost effective for low numbers (1–20) of samples. However, it has a lower throughput, detection limit (15–20%) and ability to identify novel variants (so-called discovery power) compared to NGS. Thus, the Sanger method is an effective approach for variant screening studies when the total number of samples is low [167,168].

Illumina has higher mutation resolution, from large chromosomal rearrangements to single nucleotide variants, and better discovery power. This is due to the higher sequencing depth, i.e., the number of times that a given nucleotide in the genome has been read, which provides the higher sensitivity (up to 1%) of the technique. The disadvantages of NGS include the need to analyze multiple samples at once to make the method less time consuming and expensive and the provision of shorter fragments (usually 150–300 bp, depending on the sequencing platform used) [167,169]. Therefore, NGS is a suitable technique for analysis where the massive sequencing of fragments per run or the performance of deeper sequencing to detect novel or rare variants is required.

Other NGS platforms that is possible use for fish species identification include Ion Torrent [170,171] and pyrosequencing [172,173].

Thermo Fisher Scientific’s Ion Torrent sequencing platforms perform NGS by measuring pH changes across millions of wells on a semiconductor chip during a sequencing run. Ion torrent instruments exploit the fact that addition of a dNTP to a DNA polymer releases an H+ ion. The pH is detected in each of the wells, as each H+ ion released decreases the pH. The changes in pH make it possible to determine whether that base, and how many thereof, has been added to the sequence read. Read lengths are up to 600 bp depending on the Ion Torrent system used.

Pyrosequencing is based on the “sequencing by synthesis” principle; it exploits the release of pyrophosphate from the incorporated deoxynucleotide. The pyrophosphate is converted to ATP, which is then transformed into detectable light. Pyrosequencing sequences short stretches of nucleotides, approximately 30–40 nucleotides in length, with high efficiency and accuracy [165,173,174].

Both of these platforms have the issue of homopolymer errors; the repetition of the same base in a sequence is difficult to define.

Currently, metabarcoding, a specific strength of the NGS, is coming to the fore in species identification. Unlike DNA barcoding focusing on one taxa (Section 3.4.1), metabarcoding aims to simultaneously identification of all species in a sample. Thus, more universal genes, e.g., 18S rDNA, tubulin or mitochondrial COI, 12S and 16S rDNA, are used for this purpose. The obtained amplicons are sequenced by high-throughput NGS and analyzed. Thus, a huge amount of sequence data, containing a large amount of information about a complex sample, is obtained. Thanks to this, metabarcoding has a high potential for food analysis, but eDNA is also often used to monitor the occurrence of fish species at a given location [175,176,177,178,179].

#### 3.3.3. DNA Hybridization

Nucleic acid hybridization is based on the specific association (hybridization or renaturation) of complementary nucleotide sequences derived from different molecules of DNA. The basis of hybridization is usually a fluorescently or radioactively (e.g., ^32^P) labeled probe with a known nucleotide sequence that allows the detection of a complementary sequence. Hybridization is most often performed on carriers (Southern blotting), but hybridization to nucleic acid in intact cells (“in situ” hybridization) is also possible [180,181].

DNA–DNA hybridization is still an important method used in the analysis of evolutionary relationships between organisms. The method uses the reassociation of ssDNA fragments, where heteroduplexes can be formed in a mixture of DNA of different species. Reassociation conditions (e.g., salt concentration, temperature, viscosity, fragment size) affect the possibility of hybrid molecules formation. Under strict conditions (low salt concentration, high temperature), only very similar sequences can be joined; as the conditions are gradually relaxed, more distinct sequences can pair. The greater evolutionary distance between species leads to more differences in the sequences of their DNA. Therefore, heteroduplexes of less related species form worse and dissociate more easily, respectively, in the temperature gradient. As a result, hybrid molecules can be distinguished from homoduplexes based on their different melting temperatures.

The disadvantage of the DNA–DNA hybridization method is that it does not provide information about individual features, e.g., nucleotides, or their positions in the DNA strand. In contrast, the undeniable advantage of this method is the huge range of genome sections analyzed.

#### 3.3.4. Methods Using Restriction Enzyme Cleavage

Restriction enzyme digestion methods, such as RFLP and AFLP, take advantage of the presence of polymorphism and microindels, which are often associated with the formation or, conversely, the extinction of a site that is crucial for the restriction enzyme. The common basis of both mentioned methods is the digestion of the required sequences with restriction endonucleases and the subsequent analysis of the digested fragments [182,183]. An important advantage of these methods is the low cost of analysis, and the lack of emphasis on the use of more advanced tools.

##### Restriction Fragment Length Polymorphism (RFLP)

RFLP is one way to study species diversity. It refers to variations in their DNA sequences at sites recognized by restriction enzymes. Such variation results in unique patterns of DNA fragments caused by their different lengths between restriction sites. DNA fragments are separated electrophoretically on the basis of length, size or molecular weight, and then transferred to a membrane by Southern blotting. DNA fragments on the membrane hybridize with a labeled DNA probe, allowing visualization of DNA profiles. In the absence of restriction sites, no DNA cleavage occurs. RFLP thus makes it possible to detect both the absence and the presence of such sites.

With PCR expansion, the development of methods combining PCR and restriction digestion (PCR-RFLP) began. The first step in the PCR-RFLP method is to amplify the fragment containing the variation. This is followed by treatment of the PCR products with the appropriate restriction enzyme. Depending on the presence or absence of a restriction site, restriction fragments of different sizes form. Nested PCR can be used to amplify the target region to avoid false-negative results. Thanks to the use of external and nested primers in two consecutive PCRs, this method has higher specificity, and high sensitivity and efficiency. This also allows amplification of the required fragments even at low DNA concentrations [36,184,185].

PCR-RFLP has become a highly valued technique for genotyping species-specific variations. It enables the detection of intraspecific and interspecific genetic variations such as a single-nucleotide polymorphism (SNP), a multi-nucleotide polymorphism (MNP), and microindels (insertions, deletions, duplications, and combinations involving the gain or loss of one or up to fifty nucleotides).

Disadvantages include the requirements for specific restriction endonucleases and the difficulty of identifying the exact variation when several SNPs affect the same restriction site. In addition, because PCR-RFLP consists of several steps, this method is relatively time consuming [186].

##### Amplified Fragment Length Polymorphism (AFLP)

AFLP is a highly sensitive PCR-based method suitable for population studies and for finding genetic variation between closely related species. It is based on four steps: (i) restriction (specific cleavage of total DNA by two restriction endonucleases, most often MseI and EcoRI); (ii) ligation (adapters are attached to all fragments by T4 ligase); (iii) amplification (pre-selective and selective amplification to reduce the number of fragments); and (iv) fragment visualization. AFLP requires initial screening to find the optimal primer combination; specific primers are complementary to the adapters and have an overhang of one to three bases within the studied fragment. Obtained fragments can be separated by electrophoresis on a polyacrylamide gel and visualized autoradiographically. The final visualization can also be performed in an automatic sequencer if fluorescently labeled EcoRI primers are used.

The method reflects the variability across the entire genome, and no prior knowledge of the organism being studied is required, as the primers are a complement to the adapter sequences, which is a significant benefit over other methods. Furthermore, it enables the analysis of multiple loci simultaneously. However, this can also be a disadvantage, because it is impossible to detect which fragment belongs to a particular DNA locus. Aside from this, AFLP is a relatively complicated and expensive method. Another disadvantage is the need for high-quality DNA as an input, providing fragments with different intensities, fragment homologies (which is more likely in more similar taxa), and unknown origin band origins in the obtained pattern. On the other hand, the method provides a highly reproducible pattern with a high degree of polymorphism (up to 100 fragments per primer combination), which enables the determination of intra- and inter-population variability and diversity, and the performance of a phylogeographic study [67,88,187,188,189,190]. Nevertheless, the AFLP method has been surpassed by the development of PCR and sequencing methods.

#### 3.3.5. Polymerase Chain Reaction–Single-Strand Conformation Polymorphism (PCR-SSCP)

In PCR-SSCP analysis, the target sequence is amplified and radiolabeled with primers or nucleotides. Subsequently, the amplified fragments are denatured and subjected to polyacrylamide gel electrophoretic analysis under non-denaturing conditions. Alternatively, the products are visualized on a gel using silver staining. Single-stranded DNA (ssDNA) tends to collapse into a spatial structure due to internal complementarity. Even a very small change in sequence can cause a different structural arrangement. Depending on the conformation of the ssDNA molecule, it migrates through the gel at different speeds during electrophoresis. The efficiency of SSCP decreases with increasing length of the analyzed fragment; the highest efficiency of the method is for up to 200 bp [36,191].

This method is sensitive to several conditions, such as temperature, gel concentration, and the buffer used. Therefore, the reference samples need to be analyzed in each run. The advantage is that even very similar species can be distinguished because even a very small change in the sequence will allow their distribution in the gel depending on the different mobility. This method has been used, inter alia, to identify salmon, trout, cod, and eel [36,192].

#### 3.3.6. Random Amplification of Polymorphic DNA (RAPD)

As the name of the method suggests, RAPD amplifies random sections of DNA. This is a significant difference compared to classical PCR, where a predetermined target fragment is amplified. Another difference is that the RAPD method uses only one short primer prier for amplification, usually 10 nucleotides long. This arbitrary primer often serves as both a forward and reverse primer, and during PCR amplification anneals at random sites in DNA. Variations in the genetic code give rise to unique patterns of DNA fragments in individual species. The obtained amplicons are analyzed by gel electrophoresis. The profile of the unknown sample is then compared with the profiles of species-specific bands, the so-called DNA fingerprints of the species, obtained with the same primer.

The advantages of this method are the relatively low cost, the speed of analysis, and the need for a only small amount of DNA. Furthermore, there is no need to know the fish genetic composition in advance, primers are commercially available, and both intra- and interspecies differentiation is possible. On the other hand, the application of RAPD to mixed samples or products containing highly degraded material may be problematic [36,67,193,194]. DNA degradation can cause the loss of some larger fragments in a species-specific DNA fingerprint, and thus cause incorrect species identification. Another problem is the risk of spurious matching in species producing PCR fragments of similar lengths [193,194]. Therefore, to date, PCR-RAPD has mostly been used to map out population genetics, rather than for species identification and the detection of commercial fraud [195].

#### 3.3.7. Loop-Mediated Isothermal Amplification (LAMP)

LAMP is a gene amplification method that combines speed, high specificity, and simplicity. Due to the constant temperature amplification, the LAMP method, unlike other methods, does not require expensive laboratory equipment. The use of LAMP can thus greatly simplify routine analysis, although primer design is more complex than for PCR. The method uses a combination of two or three primer pairs (outer F3, B3; inner FIP, BIP; loop primers), which allows the reaction to possess high specificity. Both outer and inner primer pairs are necessary for the initial amplification, but only inner primers are important for the cyclic amplification and elongation of emerging DNA. The amount of the target sequence is thus tripled during each half of the cycle. The result of the reaction in the presence of target DNA are lamplicons of various lengths. Loop primers are not necessary for the reaction to work properly, but they increase the rate of exponential amplification, thus reducing the time required for analysis by up to half (approximately 30 min). Unlike the PCR method, which uses a wide range of polymerases, the LAMP method generally recommends Bst polymerase, which has a higher tolerance to inhibitors. Notomi et al. [196] also recommend BcaBEST DNA polymerase (TaKaRa) when less than 10–23 moles of target DNA is used, or Z-Taq DNA polymerase when DNA polymerase needs to be added before a thermal denaturation of the DNA. Amplification products can be detected, similar to PCR, on an agarose gel. Another possibility is to use specific dyes that change their color and/or provide a fluorescent signal. Examples of such dyes are hydroxynaphthol blue, Sybr green, ethidium bromide, and calcein. During amplification, magnesium pyrophosphate is formed, leading to the formation of turbidity, which can be detected by turbidimetry [117,118,196,197,198].

#### 3.3.8. Multi-Analyte Profiling (xMAP)

xMAP technology can be used for high-throughput multiplexing and the simultaneous detection and quantification of a large number of different analytes in a single reaction. Various biomarkers, such as proteins, nucleic acids, and polysaccharides, can be analyzed, as indicated by the “x” in the method name. xMAP is based on the same principles as PCR, ELISA, and flow cytometry. The fluorescence of molecules bound to polystyrene or magnetic microspheres labeled with up to five hundred different fluorescent dyes (so-called spectral code) is measured. A target molecule is bound to each type of microsphere, which can be, inter alia, a DNA probe or an antibody. The beads are read individually using an xMAP instrument. In each of them, the fluorescence is measured after excitation with two lasers in a silica glass cuvette. The first laser excites the red fluorochrome in the bead at 635 nm, thus determining the spectral code, i.e., the type of analyte. The second laser excites the fluorochrome phycoerythrin at 532 nm; the fluorescence intensity is measured, and the amount of analyte can be determined [199]. Over the range of 3–500 analyzed targets, xMAP provides high specificity and sensitivity. Thus, very fast identification of a huge number of diverse targets can be performed, which is especially advantageous for routine analyses [200,201]. However, routine applications of xMAP are limited by its expensiveness compared to other methods.

As for current applications of xMAP for DNA analysis, methods based on DNA hybridization, sequence-specific enzymatic reaction, oligonucleotide ligation reaction, multiplex oligonucleotide PCR assay, allele-specific primer extension, or single-base chain extension can be used for multiplex detection and the quantification of nucleic acids in examined samples using xMAP technology. Thus, it can be applied, for example, for monitoring gene expression, the detection of SNPs and specific sequences, or monitoring of genes involved in the development of genetic diseases [202,203]. xMAP technology was also successfully used for the identification of fish based on the parvalbumin, COI, 16S rRNA or 12S rRNA genes [200,204,205]. However, as it is not yet widely used, its comparison with other methods is limited.

### 3.4. Data Analysis

A combination of PCR with subsequent sequencing of the products is often used to identify species. Using this procedure, primer annealing in the expected gene region can be verified, and other nucleotide sequence analyses can be performed, e.g., the detection of nucleotide variations. Methods using this principle include forensic informative nucleotide sequencing and DNA barcoding [3,61,206]. A significant advantage of these methods is that only one universal pair of primers, specific for a selected marker, can be used for multiple animal species. Among the suitable markers, the mitochondrial genes cytb and COI are widely used for fish species identification [133,207]; thus, their sequences are well known, and primer design is relatively fast. On the other hand, complex databases of reference sequences and the amplification of relatively long stretches of DNA (compared to qPCR, for example) are needed for the identification of species in processed products. Additionally, sequencing makes analysis more expensive and time-consuming, and its reliability is still being discussed [3,55].

#### 3.4.1. DNA Barcoding

Within the sphere of DNA-based fish species identification approaches, a great deal of attention has been devoted to DNA barcoding, which relies on sequence variations within a short and standardized region of the genome. This selected region, designated a “DNA barcode”, is amplified by PCR and then sequenced.

The barcoding method takes advantage of the high rate of mutations in mtDNA, which leads to a divergence of mtDNA between species and at the same time small differences in DNA within one species [60,208]. Currently, the mitochondrial genes coding COI and cyt*b* are the most commonly used because they are considered reliable DNA barcodes for the discrimination of animal species [61,103,155,208,209]. Additionally, the Consortium for the Barcode of Life (BOL; www.ibol.org, accessed on 20 October 2022) is focused on COI gene sequencing to create a barcode database for all eukaryotic species to standardize the species identification process; the selected reference sequence is about 650 bp in length for most species group [61,208]. A part of this project, the above-mentioned FISH-BOL (Section 3.1), focuses only on the identification of fish species [210].

The functionality of DNA barcoding for fish species identification has been verified in many studies [62,155,211,212,213]. However, this method also has certain limitations. The most serious disadvantage is that it cannot be used for the identification of fish in mixed samples and, further, as with all mitochondrial markers, the inability to quantify the fish DNA content in the sample. When using markers with a longer sequence (>600 bp), identification in processed products can also be problematic due to DNA degradation. Sequencing reliability is also still being discussed [66]. On the other hand, it is a dependable, fast, and cost-effective way to identify fish based on DNA analysis. For the field of ichthyology, FISH-BOL is a powerful tool for a better understanding of the natural history and ecological interactions of different fish species [210,214,215].

#### 3.4.2. Forensically Informative Nucleotide Sequencing (FINS)

The FINS technique, which combines DNA sequencing and phylogenetic analysis, uses a similar principle to that of DNA barcoding. The amplified specific DNA fragment is sequenced, and this informative nucleotide sequence is subsequently identified by phylogenetic analysis using a database of reference sequences which should be obtained from properly identified fresh samples or authentic preserved samples [216]. The results of the analysis are displayed as a phylogenetic tree, where sequences of the same species are grouped into clades. Based on the calculated distances from the reference sequences of known species, unknown species are also included in the phylogenetic tree. This method thus allows the detection of new unexplored species [217]. To achieve high-resolution results, DNA regions with high inter-specific, but low intra-specific, variation are essential. Thus, rapidly evolving regions with many informative sites in DNA sequences, for example, cyt*b*, COI, or 16S rRNA, are usually used as genetic targets [216,218].

### 3.5. Advantages and Limitations

All of the above-mentioned methods differ in many aspects and have specific advantages and limitations. The following paragraph summarizes the main characteristics of the methods.

The methods using restriction cleavage (RFLP, AFLP) benefit from their simplicity, reliability and robustness. However, the high risk of incorrect identification needs to be taken into account due to the insufficient study of related species, which may form the same restriction profile or polymorphism in the analyzed DNA, leading to a change in the restriction site and thus incorrect evaluation of the analysis. As a result, some authors tend to recommend the use of sequencing and phylogenetic analysis techniques [64,219], such as DNA barcoding or FINS. These methods provide a high power of diagnosis, which minimizes the risk of misidentification of the species. When analyzing a species that has not been studied yet, it will be included in the appropriate node in the phylogenetic tree based on its nucleotide sequence. The phylogenetic tree also contains reference sequences of several species; the assignment to a node corresponding to a certain species thus allows the identification of blank samples [217]. However, sequencing-based methods run into limitations in the analysis of processed and/or pooled samples. In this case, species-specific PCR or LAMP seems appropriate. The advantages of the LAMP method compared to PCR are the lower demand for lab equipment and the possibility of shortening the amplification time by up to a third [220], but PCR is a more robust method. PCR and FINS also have high potential for building nucleotide databases. However, the above-mentioned methods (PCR, LAMP, FINS, RFLP) require prior knowledge of the DNA sequence of the analyzed species for the analysis itself, unlike RAPD and AFLP, for example. Next, RAPD and AFLP can be used for the analysis of multiple loci at once. AFLP markers also exhibit much higher variability than, for example, isozymes. However, neither method is very robust against DNA degradation, and they both have low potential for interlaboratory reproducibility. Compared to AFLP and RFLP, RAPD is considered the most reliable method for species identification when the genome sequence is unknown [67,221]. However, for both RAPD and RFLP, intraspecific variation can be problematic, in contrast to the SSCP method, for example. On the other hand, SSCP is more demanding and always requires the presence of a reference sample on the gel together with the analyzed samples. Despite the high sensitivity of SSCP or PCR-SSCP and its capability of intraspecific differentiation, the amount of information obtained from these methods is much lower than with sequencing [30,192,193,222].

A brief overview of the comparison of the methods is given in Table 2.

### 3.6. Evidence of Adulteration in Fish by DNA Analysis

The proper labeling of food and food products is important not only for fair trading, but above all for consumer protection. Nevertheless, adulterated products continue to spread in today’s markets. Fish and seafood are some of the products that are widely tampered with to bring down the high price of the raw material. With fish, species replacement, the undeclared addition of water to muscle, or declaring thawed fish to be fresh are the main forms of adulteration found [218,223,224].

Molecular methods for identifying fish species are based on the detection of DNA polymorphism. Therefore, it is necessary to select a segment of DNA that can be detected even in highly processed food products. The amplification of such target DNA sequences by PCR has become popular. PCR is used to determine the DNA present in fresh or frozen meat, but also in processed and multispecies products [33,47,225].

For example, Keskin and Atar [226] verified the presence of the Alaskan cod (*Theragra chalcogramma*) in surimi products. Using COI gene analysis, they determined that only 16% of the products corresponded to the declared species. Next, Leonardo, Nunes, Monteiro, Conte-Junior, Del Aguila and Paschoalin [91] tested the authenticity of sardines on the market in the state of Rio de Janeiro. They found that 40% of the tested products had been replaced. The authenticity of fish species was verified by phylogenetic analysis using the cyt*b* gene. Fraudulent samples were identified as *Clupea harengus*, *Brevortia aurea*, *Centengraulis edentulus* and *Scomber japonicus*. These species are cheaper and contain 40% less protein than authentic sardines. Along with sardines, European anchovies (*Engraulis encrasicolus*) are one of the most commonly adulterated items in fish products. However, anchovies are particularly suitable in the Mediterranean diet of children and the elderly thanks to their high content of polyunsaturated fatty acids. Their adulteration can thus lead to a disturbance of the diet. Therefore, Pappalardo and Ferrito [114] analyzed 50 seafood products by PCR-RFLP method to evaluate the species identity. 14% of the products were found to be mislabeled; *Engraulis japonicus*, *Sardinella aurita* and *Sardina pilchardus* were present.

PCR-RFLP has also been successfully used to identify members of the Cyprinidae family. Chen, Hsieh and Hwang [95] found nearly 38% of processed Cyprinidae commercial samples to be adulterated; using the FINS method, it was confirmed that *Oreochromis* spp. were used as substitutes.

Furthermore, Herrero et al. [123] determined that for 5% of the salmonid products analyzed, the name of the species on the label did not match the real species. They also confirmed the results by means of sequencing and with the PCR-RFLP method developed by Espiñeira, Vieites and Santaclara [133]. Both methods were in agreement on the actual fish identity. Verification of salmonids was also addressed in studies of Xiong et al. [117,118], where primers designed specifically for the sequence of cyt*b* and COI genes were used to identify Atlantic salmon (*Salmo salar*) in commercial fish products. First, the 29 samples declared to be Atlantic salmon were analyzed using the LAMP method. It was found that only six samples (20%) matched the label [117]. One year later, they used duplex qPCR combined with melting curve analysis for the simultaneous detection of Atlantic salmon and rainbow trout (*Oncorhynchus mykiss*). As in the previous case, 80% of mislabeled products were detected [118]. These results were also confirmed via DNA-barcoding in both cases. DNA barcoding was also used for analysis by Panprommin and Manosri [227], who focused on the analysis of fish fillet products in Thailand. They showed that salmon and trout had been adulterated. In fish products, they noticed *S. salar* being mistaken for *O. mykkis* and *O. kisutch*.

The mini-barcoding method, focusing on shorter mtDNA fragments (100–200 bp), has also been successfully verified for the authentication of fish products. In processed food, this procedure is more successful than using DNA encoding standard 650 bp DNA fragments [47,56].

**Figure 3:**
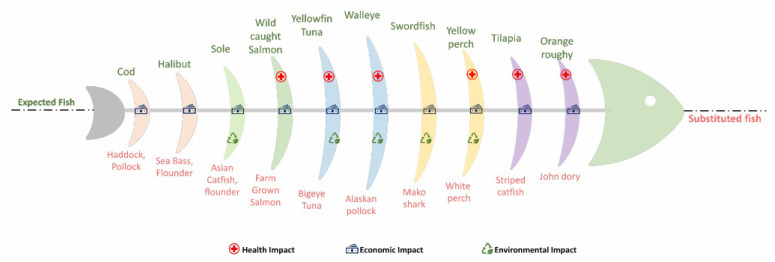
Commonly substituted fishes and their impacts [228,229,230,231].

An example of a frequently substituted species and the impact of their adulteration is shown in Figure 3.

Many authors have also considered the possibility of distinguishing between fresh and thawed meat using DNA analysis. The ability to identify thawed fish declared to be fresh using DNA-based methods is given by the fact that during the freezing and subsequent thawing of meat, enzymes are released from the cells, and thus DNA molecules are degraded by endonucleases and exonucleases. Thus, DNA damage can be detected using a Comet assay [232]. However, analytical methods other than DNA-based ones, especially enzymatic, spectroscopic, bioimaging or sensory techniques, or combinations thereof, are still more appropriate to demonstrate the declared labeling of thawed fish as fresh or the addition of water to the muscle [232,233].

### 3.7. Detection of Genetic Modifications in Fish

With the development of modern biotechnology, progress is also being made in the preparation of transgenic (with an altered genome containing foreign DNA) and gene-edited (with altered genome without foreign DNA introduction) fish. Fish is an excellent animal model for genetic research because of their characteristics—mainly fast growth rate, high fecundity, and in vitro fertilization [234].

The first transgenic fish, rainbow trout (*Oncorhynchus mykiss*), was produced in 1984 by Maclean and Talwar [235]. Since then, various fish species have been genetically modified (GM). The GloFish^®^, an aquarium fish prepared by inserting a plasmid with a gene for a fluorescent protein of different colors (GFP—green; RFP—red; YFP—yellow) with a strong muscle-specific promoter, is probably the best-known example of transgenic fish. It is not an organism intended for human consumption and was not subject to restrictions on the breeding of GM fish by the Food and Drug Administration (FDA) [236,237]. Nevertheless, even these fluorescent fish are adulterated; it is not uncommon for various fish species to have a dye incorporated into the body by injection or contained in the feed. However, unlike the real GloFish^®^, these fish lose their color after a few weeks (http://www.practicalfishkeeping.co.uk, accessed on 20 October 2022). Therefore, it is necessary to have methods capable of confirming or refuting the presence of specific transgenic DNA in samples as well as reliable methods for fish species identification, which form part of fish GMO detection/relative quantification. Such methods are being developed, and the majority of them are based on the targeted approaches that require knowledge of the specific GMO. For the detection and quantification of GMOs, the qPCR is the most widely used method. Recently, however, the use of amplicon or genome sequencing has also been expanding (see Table 3). The qPCR multi-step approach used for GMO detection and quantification includes several following PCR amplifications. The first step is a species-specific PCR to verify the amplifiability of DNA used. This is followed by screening PCR for the detection of promoters, terminators, or inserted genes (COI, gene of interest). The next step is the detection of inserted transgenic cassettes and/or event-specific amplification (necessary, for example, in the EU for control purposes). However, organisms prepared by gene editing (CRISPR, zinc finger or TALE nucleases) harboring small changes, e.g., base mutation, without inserting a transgenic cassette are very difficult to detect. Using targeted PCR or sequencing, a change in the DNA sequence of the analyzed organism can be demonstrated, but currently used detection methods cannot reliably demonstrate the process of DNA sequence change (spontaneous mutation or the work of molecular biologists). Thus, event-specific fish detection is possible for GM fish prepared by insertion of the transcription cassette and for gene-edited organisms with a known unique DNA change.

A PCR method for the differentiation of non-transgenic and transgenic ornamental fish was suggested by Rehbein and Bogerd [238], and a qPCR to detect ornamental transgenic fish harboring green, yellow, and red fluorescent coloring was recently proposed by Debode, Marien, Ledoux, Janssen, Ancion and Berben [236].

The first transgenic animal approved for human consumption was the AquAdvantage Salmon^®^ (in 2015, USA) [239,240]. GM salmon, like some other GM fish (tilapia, trout, carp, bream), can grow to market size much faster than ordinary species. This is usually achieved by inserting a transgene cassette containing, e.g., the promoter from fish antifreeze protein gene (AFP), together with cDNA coding the fish growth hormone (GH). Debode et al. [241] published two detection methods using qPCR for food-important fish species, one for AquAdvantage^®^ Atlantic salmon and the second for GM coho salmon (*Oncorhynchus kisutch*; for research purposes developed by Fisheries and Oceans Canada) [241]. For species-specific amplification, primers complementary to exon 5 of GH (nuclear) specific for salmonid species were used. The sequence of the growth hormone genes has also been used in other studies detecting GM fish [242,243]. In a study by Masri, Rast, Ripley, James, Green, Jia and Devlin [242], the DNA was not only isolated from the fish muscle of transgenic Pacific Coho Salmon (*Oncorhynchus kisutch*), which is a primary source of DNA in a majority of analyses, but DNA from fins, scales, bones, eggs, skin, slime, and blood was also been successfully amplified.

Nowadays, advanced molecular biological techniques such as TALEN or CRISPR are used for fish DNA editing. Thus, various GM fish species are beginning to appear in the world. GM red sea bream (*Pagrus major*), known under the Japanese name “Madai”, was launched in Japan in 2021 and is very popular in the area. The CRISPR method was used for a knockout of the myostatin protein. This resulted in a significant increase in consumable muscle in GM red sea bream compared to conventional types. Its breeding could thus be part of strategies to ensure sufficient food for the world’s growing population [244]. Furthermore, gene-edited tilapia, labeled as FLT-01, was developed by Intrexon (subsidiary AquaBounty Technologies). This tilapia has a 26-bp deletion in its myostatin gene, which leads to an improvement in feed conversion ratio and an increase in growth [245].

Affecting muscle growth is probably the most common modification in fish. However, there are already other transgenes that have significant phenotypic effects when introduced into fish. For example, a tolerance to cold temperatures of Atlantic salmon caused by a transgene antifreeze protein, bacterial disease resistance in carp or catfish thanks to lactoferrin or cecropin, or affecting carbohydrate or vitamin C metabolism in rainbow trout (glucose transporter, hexokinase, L-gulono-gamma-lactone oxidase) [246].

These GM and gene-edited fish are not currently permitted for breeding or for consumption in many countries of the world, including the European Union, even though with ever deeper knowledge of fish genomes and more advanced methods of molecular biology, such as the above-mentioned CRISPR, it is becoming easier to develop modified transgenic event fish. Therefore, reliable methods for monitoring for their unauthorized presence in the markets of these countries, including fish species identification at least to verify the quality and quantity of DNA before GM analysis, are needed.

**Figure 4:**
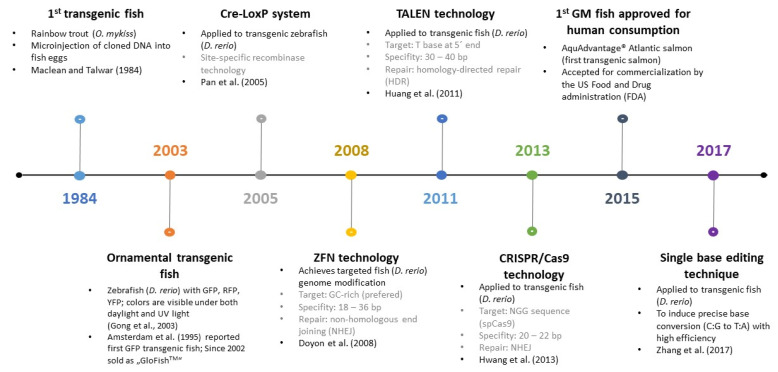
Major milestones in genetically modified fish research [235,247,248,249,250,251,252,253].

An overview of significant milestones in GM fish research is shown in Figure 4; an overview of developed GM fish and/or their detection methods is given in Table 3.

**Table 3:** Summary of current GM fish developed for possible commercial purposes and methods of their detection.

GM Fish	Purpose of Modification	Detection Method	DNA Marker (Used Gene)	Transgen/Modification	References
Atlantic salmon (*Salmo salar*)	To be more efficient for aquaculture compared with conventional non-GM Atlantic salmon	qPCR	Growth hormone gene 1 (GH1)	AquAdvantage^®^; inserting the opAFP-GHc2 transgene construct (EO-1α) into the nuclear DNA of *S. salar*, which includes a single copy transgene cassette coding a Chinook salmon (*O. tshawytscha*)-derived GH gene driven by an antifreeze protein promoter from the ocean pout (*Z. americanus*)	Soga et al. [254]
	Encourages growth rates	qPCR	Growth hormone gene 1 (GH1)	Aquadvantage^®^; GH transgenic *S. salar* contain a gene construct (opAFP-GHc2; EO-1α) consisting of GH cDNA from *O. tshawytscha* that is regulated with antifreeze protein gene sequences from an *Z. americanus*	Hafsa, Nabi, Zellama, Said and Chaouachi [243]
	Growth enhancement	PCR	Growth hormone gene (GH)	Microinjection of “all fish” chimeric GH gene construct in eggs: an antifreeze protein gene (AFP) promoter from ocean pout (*Zoarces americanus*) linked to a chinook salmon (*O. tshawytscha*) GH cDNA clone; AFP promoter is active and suitable for gene transfer in salmonids; in the future called as ”AquAdvantage Salmon^®^“	Du et al. [255]
	Resistance to very cold water	PCR, immunoblotting	Antifreeze protein gene (AFP)	Fertilized Atlantic salmon eggs were injected through the micropyle with winter flounder antifreeze protein gene under the control of its natural promoter	Shears et al. [256]
Atlantic and Coho salmon (*Salmo salar*, *Oncorhynchus kisutch*)	AquAdvantage^®^ Atlantic salmon: commercial purposes (encourages growth rates to generate fast-growing strains for potential use in aquaculture); coho salmon for research purposes (to study the physiology and behavior of transgenic salmon)	qPCR	Growth hormone (GH)	AquAdvantage^®^ Atlantic salmon: Antifreeze Promoter and Terminator (*M. americanus*), GH gene from *O. tshawytscha* Transgenic Coho salmon: Promoter (Metallonein), Growth hormone gene and Terminator are from *O. nerka*	Debode, Janssen, Marien, Devlin, Lieske, Mankertz and Berben [241]
Coho salmon (*Oncorhynchus kisutch*)	To generate fast-growing strains for potential use in aquaculture (modification allow expression of GH from all tissues in the salmon; elevate circulating levels of the growth hormone in the blood of the GM fish)	PCR	Growth hormone gene 2 (GH2)	GH coding regions in these transgenes have been fused to the sockey salmon (*O. nerka*) metallothionein-B gene promoter; DNA construct allow expression of GH from all tissues in the salmon	Masri, Rast, Ripley, James, Green, Jia and Devlin [242]
Common carp (*Cyprinus carpio*)	For research purposes (for studying integration, expression and inheritance of foreign genes in this species); Improve fish breed	Survived embryos (after 60 days) were screened by PCR	Mouse metallothionein-I promoter (mMT-I)/human growth hormone gene (hGH)	Microinjection of the human growth hormone gene (hGH) into the germinal disc of common carp one-cell embryos	Hernández et al. [257]
	For research purposes; Improve fish breed	Dot blot and Southern blot hybridization, using the RSV-LTR and/or the GH cDNA sequences as probes; Expression of the trout GH polypeptide was detected by immunobinding assay	Growth hormone gene (GH)	Microinjection of the recombinant plasmid containing the Rous sarcoma virus-long terminal repeat (RSV-LTR) promoter linked to rainbow trout (*Salmo gairdneri*) growth hormone (GH) cDNA; “All-fish” constructs	Zhang et al. [258]
Goldfish (*Carassiusauratus*)	Rapid growth		Growth hormone (GH)	Pronuclear microinjection of recombinant plasmid pBPVMG-6; the mouse metallothionein-1 (MT-1) promoter was fused with the human growth hormone (hGH) gene inserted into a bovine papillomavirus vector (pBR-BPV)	Zhu et al. [259]
Northern pike (*Esox lucius*)	Improve fish breed; for research purposes	Southern hybridizations of tissues from a microinjected individuals	Growth hormone (GH)	Microinjection of bovine (bGH) or chinook salmon (csGH) growth hormone cDNA genes	Gross et al. [260]
Red sea bream (*Pagrus major*)	Increase of skeletal muscle mass and reduced body length	Verification of genome editing: sequencing of target region in fish muscle, brain, liver or gonad; morphological changes	Myostatin (Pm-mstn)	genome editing: CRISPR/Cas9 (microinjection of the Cas9 RNA and sgRNA was used to introduce the CRISPR/Cas9 system); deletions in the first exon of the Pm-mstn, which cause disruption of the C-terminal active domain of MSTN	Kishimoto, Washio, Yoshiura, Toyoda, Ueno, Fukuyama, Kato and Kinoshita [244]
	Optimization of microinjection parameters as important step for successful genome editing; tested on myostatin because it is known that its deficiency does not affect the viability of fish	Verifying the success of the microinjection and the effects of the three tested factors were estimated by the survival rate (38–40 h post fertilization)	Myostatin	In vitro fertilization and microinjection of the Cas9 RNA (100 ng/µL) and sgRNA (50 ng/µL) mixture	Kishimoto et al. [261]
	Increase of skeletal muscle mass; research purpose		Myostatin (*Pm-mstn*)	myostatin complete knockout (CRISPR/Cas9)	Ohama et al. [262]
Tiger pufferfish (*Takifugu rubripes*)	Optimization of microinjection parameters as important step for successful genome editing; tested on myostatin because it is known that its deficiency does not affect the viability of fish	Verifying the success of the microinjection and the effects of the three tested factors were estimated by the survival rate (6–7 days post fertilization)	Myostatin	In vitro fertilization and microinjection of the Cas9 RNA (100 ng/µL) and sgRNA (50 ng/µL) mixture	Kishimoto, Washio, Murakami, Katayama, Kuroyanagi, Kato, Yoshiura and Kinoshita [261]
Rainbow trout (*Oncorhynchus mykiss*)	To improve desirable genetic traits such as growth		Growth hormone (GH)	Pronuclear microinjection into newly fertilized rainbow trout; Mouse metallothionein gene within *E. coli* plasmid pBR 322	Maclean and Talwar [235]
	To enhance disease resistance	PCR; (RT)-PCR analysis was used for detection of expression of cecropin P1 and CF-17 transgenes	Cecropin P1 or synthetic cecropin B analog (CF-17) gene	Electroporation; expressing cecropin P1 or a synthetic cecropin B analog, CF-17, transgene by sperm-mediated gene transfer method	Chiou et al. [263]
Rainbow trout and Super mud loach (*Oncorhynchus mykiss*, *Misgurnus mizolepis*)	To be more efficient for aquaculture compared with conventional non-GM fish	PCR, qPCR	Growth hormon (GH)	Modified target genes:metallothionein (MT) and mud loach chloramphenicol acetyltransferase (MLcat);	Chae et al. [264]
Tilapia (*Oreochromis niloticus*)	Improve fish breed (commercial applications)	CAT (chloramphcnicol acetyl transferase) assay was used to test for gene expression in the transgenic fish	Mouse metallothionein-I promoter (mMT-I)/rat growth hormone gene (rGH)	Microinjection of transgenes; injected DNA constructs comprising a carp beta-actin promoter sequence spliced to the bacterial reporter CAT gene	Rahman and Maclean [265]
	For research purposes (studying integration and expression of foreign genes in fish)	Southern blot and dot blot analysis	Growth hormone gene 1 (GH-1)	Microinjection: EcoRI-DNA fragment containing the mouse metallothionein-I promoter (mMT-I) fused to a structural gene coding for the human growth hormone (hGH) was injected into the germinal disc	Brem et al. [266]
Zebrafish (*Danio rerio*)	For research purpose (fluorescent protein used as a reporter)	Epifluorescence microscopy, PCR	Green, fluorescent protein gene (GFP)	Microinjection of GFP cDNA (*KpnI-Sac*I fragment from TU65) insterted into a pXex vector (composed of the enhancer sequence, promoter and 5′ untranslated sequence from the Xenopus ef1α gene)	Amsterdam et al. [252]
	For research purposes (fluorescent protein used as a reporter); Two-color transgenic Zebrafish	fluorescence microscope	Green, red fluorescent protein gene (GFP, RFP)	Microinjection of more different DNA constructs (CK-EGFP, pCK -RFP, pMLC-EGFP, and pMLC-RFP) into each embryo; promoters: keratin8 gene (krt8) for skin specificity, myosin light chain 2 gene (mylz2) for muscle specificity; *gfp* and *rfp* reporter gene constructs, pEGFP-1 and pDsRed-1	Ju et al. [267], Wan et al. [268]
	Ornamental (fluorescent colors), bioreactor (system for production of recombinant proteins)	Fluorescence (visible to unaided eyes under daylight and ultraviolet light in dark); level of protein expression was estimated by SDS–polyacrylamide gel electrophoresis; Northern blot hybridization. Was used for analysis of transgenic and endogenous RNA expression	Green, yellow or red fluorescent protein gene (GFP, YFP, RFP)	Microinjection of a construct pMYLZ2-EGFP, pMYLZ2-RFP, and pMYLZ2-YFP into embryos (at 1- or 2-cell stage); gene coding for a green fluorescent protein (GFP) and yellow-orange fluorescent protein (YFP) originally comes from a bioluminescent jellyfish (*Aequorea Victoria*), red fluorescent protein (RFP) comes from anemone (*Discosoma sp*.); the gene is expressed under the transcriptional control of the strong muscle-specific promoter of the *myosin light peptide* 2 gene *(mylz2)*; plasmid contein 2kb *mylz2* promoter	Gong, Ju and Wan [237], Gong et al. [253]
	Site-directed recombination in transgenic fish	PCR (To confirm the excision of transgene)	*gfp* gene	*Cre/loxP* system; a floxed (loxP flanked) *gfp* (*green fluorescent protein*) gene construct under the muscle-specific *mylz2* promoter; in vitro synthesized Cre RNA was injected into transgenic zebrafish embryos	Pan, Wan, Chia, Tong and Gong [247]
Zebrafish, Tetra (*Danio rerio*, *Gymnocorymbus ternetzi*)	Ornamental (fluorescent colors under UV light)	qPCR, fluorescent microscopy (both methods allow to distinguish artificially colored fish from a GM fluorescent fish)	Green, yellow or red fluorescent protein gene (GFP, YFP, RFP), cytochrome-c-oxidase subunit III (COIII), tRNA-Gly and ND3	Glofish: Microinjection of a gene coding for a fluorescent protein	Debode, Marien, Ledoux, Janssen, Ancion and Berben [236]

### 3.8. Current Trends and Future Challenges

As can be seen from the text above, many reliable methods are currently available for the detection and identification of fish DNA. The performance of PCR amplifications and restriction digests already makes them very feasible, and the protocols are successfully transferable (verifiable) between laboratories. Nevertheless, constant progress is being made in this field, which makes it possible to speed up and improve the quality of analysis, which is necessary nowadays. The continuous improvement of established methods is exemplified, for example, by modifications of primers and probes used for PCR, such as LNA (locked nucleic acid), ZNA (Zip Nucleic Acids), the Plexor™ System, etc., that positively influence the binding of oligonucleotides to the selected target section. For example, an LNA oligonucleotide has a higher affinity for DNA than a standard DNA oligonucleotide, which can increase the temperature stability of the synthesized products, as well as hybridization specificity and sensitivity. The modifications also allow the Tm values of the primers and probes to be adjusted, which may allow the multiplexing of primer sets that would otherwise not be combined due to their different annealing temperatures. With increasing demands for speed, accuracy and cost of analysis, the development of such modifications seems necessary.

Another example of the development of methods for fish DNA identification is the xMAP method. Thanks to its properties, such as excellent precision, high sensitivity, specificity, rapid data acquisition, high-throughput analysis or possibility of multiplex and simultaneous detection of different analytes [200], xMAP has the potential for feed and food testing, including fish species identification.

Rapid detection is also provided by the LAMP method. This method has already been published for several types of fish (see Table 1). However, there is definitely potential to develop an assay to detect a broader selection of fish. In recent years, there has also been an increase in articles in which fluorescence detection is used, similar to qPCR [269]. This enhancement makes it possible to remove the end-point analysis step. At the same time, it offers the possibility of the simultaneous detection of multiple target sections of DNA, which would not be possible with traditional detection on agarose gel or based on the color change of the solution or the presence of turbidity.

Thanks to technological progress, the sequencing of PCR amplicons has also expanded in recent years. Sequencing methods provide a large amount of information from a single DNA fragment; however, the disadvantage is that it is still time and financially demanding compared to other identification methods based on DNA analysis, for example the aforementioned PCR. To reduce the price and improve the availability of sequencing services for smaller laboratories, samples or PCR amplicons are often outsourced to laboratories specializing in these processes. However, even the processing of the obtained sequencing data is not completely straightforward and requires workers with a certain degree of knowledge and experience. Therefore, it is also necessary to constantly improve bioinformatics tools to make them more user friendly and to make data evaluation easier.

Another pitfall of analysis is the still limited number of annotated primary sequences of fish genomes available. This can complicate data analysis. For example, NGS-based SNP discovery is very challenging in species that do not have a reference genome, due to the misalignment of short sequence reads of different individuals and genotypes generated by current NGS technologies. We expect that this situation will be greatly improved in the near future, considering the increasing number of different platforms for DNA sequencing (Roche, Illumina, Oxford Nanopore etc.) and the interest in sequencing analysis, which will lead to more sequencing data becoming available. Additionally, the correctness of fish species identification, as well as the accuracy of SNP prediction, which generally increases with increasing reading depth, will, in our opinion, continue to improve as the parameters of the sequencing platforms themselves improve with technological progress. Thanks to the availability of a wide range of high-quality whole-genome fish sequences, it will be easier to design primers for specific representatives of fish (species-specific), but also for entire families, as well as the study of fish evolution itself.

In the analysis of food and feed, articles involving the use of DNA metabarcoding, i.e., the combination of DNA barcoding with massive parallel sequencing based on NGS methods, have appeared in recent years. The use of metabarcoding has been successfully reported to detect the adulteration of mammalian species in meat and dairy product [270]. In a study by Gense et al. [271], this approach was successfully used to identify 11 bivalve species and analyze their products. To the best of our knowledge, no study using this approach has been published for fish species identification yet. DNA metabarcoding is particularly suitable for the detection of unexpected species not detected in the analyzed samples by targeted methods such as qPCR. The price of sequencing has also been decreasing in recent years; with these facts in mind, we expect the use of metabarcoding DNA to also be extended to fish in the near future.

Another disadvantage of sequencing is the use of longer DNA fragments. Obtaining such sections with sufficient quality can be difficult, especially for fish products or otherwise processed fish muscle. As a result of modification (physical, chemical), DNA damage can occur to varying degrees, for example, mutations, indels and fragmentation/degradation. At the same time, substances can be present in the sample that can inhibit the isolation process and thus make it impossible to obtain DNA, as well as substances that elute into the solution together with the DNA and thus complicate the subsequent analysis. For this reason, we consider obtaining a sufficient quantity of high-quality DNA to be one of the key steps (and often the biggest difficulty in analyses using DNA-based methods). DNA isolation itself is not discussed in this work, as it is a complex process, and a detailed description and analysis of the advantages and/or pitfalls associated with individual isolation steps should be published in a separate review. However, we believe that although the available literature sufficiently covers the issue of DNA isolation from different parts of fish [272,273,274,275], the development of increasingly better isolation procedures is necessary. The yield and purity of the purified DNA depends on the DNA extraction and purification method, and no universal method is valid for all food matrices [276]. Improvements in extraction or purification procedures can yield higher and purer DNA. For this purpose, the development of standard materials for the control of the extraction process is also offered. A suitable standard, which would be added to the sample before the extraction itself and would go through the entire process until the end of the analysis together with the sample itself, would also allow more accurate DNA quantification. PCR inhibition could be evaluated through the effect on the internal standard material in the amplification profile. The fish meat authentication process in general would benefit from the development of a unified method to ensure the comparability of results across traded fish species.

The development of a unified methodology for easier and more efficient assessment and approval of GM fish is also necessary. A generally accepted risk assessment model for regulators could prevent (or at least limit) commercial conflicts regarding the use and marketing of GM foods. At the same time, the regulatory authorities could deal more with the specific problems of the assessed foods. This, along with educating the public, could help increase public confidence in GM foods and trust in these potentially beneficial technologies.

Current technologies have enabled the development of transgenic fish with increased body growth index [277,278,279,280], resistance to diseases or climate change [263,281,282,283], and other biotechnological applications important for research, such as the use of transgenic fish as bioindicators [253,284,285] or bioreactors [286,287,288]. We anticipate that biotechnological advances will lead to the development of techniques to generate transgenic fish with better efficiency and effectiveness than current methods. Regardless of which transgene is used, the goal should be to improve nutrient use and reduce costs for fish farming. Improvements in aquaculture could reduce fishing pressure and thus prevent the extinction of wild fish. In addition, reducing the use of antibiotics, insecticides or fungicides as a result of fish resistance to disease would reduce the environmental impact of fish farming.

Nevertheless, the safety of GM foods is still being discussed, and the sale of selected transgenic fish is therefore only approved in a few countries. Due to the globally declining stocks of wild fish and the food situation in the world, it can be assumed that transgenic fish will be sold in some countries even without the appropriate permits. Therefore, the development of methodologies enabling the necessary market control and detection of GM fish is essential.

## 4. Conclusions

Given the current worrying food situation, in particular the lack of food and the economic situation, the adulteration of food can be expected to become more frequent in the near future, including fish meat and fish products, as they comprise one of the most important commodities. At the same time, fish is one of the main sources of allergens, whose content differs among species. Therefore, fast and reliable methods of fish species identification are being sought. The selection of an appropriate method, target molecules, and identification markers are crucial for a successful analysis. Currently DNA-based methods are preferred, since both qualitative and quantitative analyses of fish meat and processed fish products can be performed with their use, and they have very high levels of specificity and sensitivity. However, the methods used can still be improved in terms of their capacity, speed, price per reaction, and laboratory availability worldwide. In addition to the established PCR method and its variants and the PCR associated with the sequencing of the amplified section (barcoding), the LAMP and xMAP methods are promising tools for fish authentication. Nevertheless, given the need to quantify the proportion of meat contained in the product, we still consider PCR to be the most appropriate method for the identification and quantification of species.

## Figures and Tables

**Table 1:** Overview of mitochondrial and nuclear DNA markers used for fish species identification.

Family	Genus	Detection Method	DNA Marker *		Declared Species Specifity	References
			Gene	Type		
Anguillidae	Eel (*Anguilla)*	PCR-RFLP, PCR-SSCP	*cytb*	mtDNA	*A. anguilla*, *A. australis*, *A. rostrate*, *A. japonica*	Rehbein et al. [87]
		AFLP	- (genomic DNA)	nDNA	*A. anguilla*	Maldini et al. [88]
Carangidae	Horse mackerel (*Trachurus*)	FINS	*cytb*	mtDNA	25 species (22 Carangidae, 1 Mullidae, 2 Scombridae species)	Lago et al. [89]
		real-time PCR	*pvb*	nDNA	*T. trachurus*, *S. scombrus*	Prado et al. [90]
Clupeidae	Sardines (*Sardina*)	PCR-RFLP	*cytb*	mtDNA	*B. aurea*, *C. harengus*, +*C. edentulous* (Engraulidae)	Leonardo et al. [91]
	Sardines, Sprat, Herrings (*Sardina*, *Sardinella*, *Sprattus*, *Clupea*)	FINS, searching in BLAST	*16S rRNA*	mtDNA	*S. pilchardus*, *S. aurita*, *S. sprattus*, *C. harengus* +Fish species from Engraulidae, Salangidae and Scombridae family	Armani et al. [92]
	Sardines (*Sardina*)	DNA barcoding, real-time PCR	*COI*	mtDNA	*S. pilchardus* *+other sardine species (identification by DNA barcoding)*	Xiong et al. [93]
Cyprinidae	Carp (*Cyprinus*)	real-time PCR	*D-loop area*	mtDNA	*C. carpio*	Bajzik et al. [94]
	Carp, Barbell, *Minnow* (*Acrossocheilus*, *Candidia*, *Carassius*, *Ctenopharyngodon*, *Cyprinus*, *Varicorhinus*, *Hemibarbus*, *Zacco*)	PCR-RFLP, FINS	*cytb*	mtDNA	*A. paradoxus*, *C. auratus auratus*, *C. barbatus*, *C. carpio carpio*, *C. idella*, *H. labeo*, *V. barbatulus*, *Z. pachycephalus*	Chen et al. [95]
	Carp, Barbell, *Minnow*, Gudgeon, Bitterling (*Acrossocheilus*, *Candidia*, *Carassius*, *Ctenopharyngodon*, *Cyprinus*, *Varicorhinus*, *Hemibarbus*, *Zacco*, *Pseudorasbora*, *Rhodeus*)	PCR-RFLP	*cytb*	mtDNA	*A. paradoxus*, *C. auratus auratus*, *C. barbatus*, *C. carpio carpio*, *C. idella*, *H. labeo*, *P. parva*, *R. ocellatus ocellatus*, *V. barbatulus*, *Z. pachycephalus*.	Chen et al. [96]
	Carp (*Cirrhinus*, *Labeo*)	RAPD	-	genome	*C.catla*, *L. rohita*, *L. calbasu*, *C. mrigala*	Barman et al. [97]
Esocidae	Pike (*Esox*)	microsatellites	7 microsatellite loci in genome		*E. lucius*	Lucentini et al. [98]
		AFLP	*COI*, *cytb*	mtDNA	*E. lucius*, *E. flaviae*	Lucentini et al. [99]
		DNA minibarcoding, PCR	*COI*, *Plagl2*	mtDNA, nDNA	*E. lucius*, *E. aquitanicus*	Denys et al. [100]
Gadidae	Cod (*Gadus*)	LAMP, PCR	*cytb*	mtDNA	*G. morhua*	Saull et al. [101]
		real-time PCR	*COI*	mtDNA	*G. morhua*	Herrero et al. [102]
		RFLP	*COI*	mtDNA	*G.morhua*, *G. macrocephalus*	Mueller et al. [103]
		SNPs	8221 loci	genome	*G. morhua*	Poćwierz-Kotus et al. [104]
		DNA barcoding, NGS	10 nuclear barcode regions	nDNA	*G.morhua*, *G. macrocephalus*	Paracchini, Petrillo, Lievens, Kagkli and Angers-Loustau [56]
		PCR-RFLP	*tRNA^Glu^/cytb*	mtDNA	*G. morhua* *+23 species from different genus*	Wolf et al. [105]
	Cod, haddock (*Gadus*, *Melanogrammus*)	LAMP	*cytb* (+*12S rDNA*)	mtDNA	*G. morhua*, *G. macrocephalus*, *M. aeglefinus*	Wang, Feng and Tian [44]
	Cod, pollock (*Gadus*, *Theragra*)	LAMP	*cytb*	mtDNA	*G. morhua*, *G. macrocephalus*, *T. chalcogramma* +two oilfish species (*R. pretiosus*, *L. flavobrunneum*)	Li et al. [106]
	Cod, haddock, Pollock (*Gadus*, *Melanogrammus*, *Theragra*)	PCR	*PanI*	nDNA	*G. morhua*, *M. poutassou*, *M. merlangus*, *P. virens*, *T. chalcogrammaz* *+Merluccius* spp.	Hubalkova, Kralik, Kasalova and Rencova [33]
		HRM	*12S rRNA*	mtDNA	*G. morhua*, *G. macrocephalus*, *G. chalcogrammus*, *P. virens*, *M. aeglefinusz* *+M. merluccius*, *M. australis*, *A. pectoralis*	Shi et al. [107]
	Cod, escolar (*Gadus*, *Lepidocybium*)	PCR-RFLP	*cytb*	mtDNA	*G. morhua*, *G. microcephalus*, *L. flavobrunneum* *+R. pretiosus*, *R. hippoglossoides*	Hwang et al. [108]
	Haddock (*Melanogrammus*)	real-time PCR	transferrin	nDNA	*M. aeglefinus*	Hird et al. [109]
Lophiidae	Angler *(Lophius*)	FINS, PCR-RFLP	*COI*	mtDNA	*L. budegassa*, *L. vomerinus*, *L. piscatorius*	Espineira et al. [110]
		RAPD	-	genome	*L. gastrophysus*	Ramella et al. [111]
		real-time PCR	*pvb*	nDNA	*L. budegassa*, *L. piscatorius*	Mukherjee et al. [112]
Merluccidae	Hake *(Merluccius*)	FINS	*cytb*	mtDNA	*M. merluccius*, *M. hubbsi*, *M. capensis*, *M. merluccius* *+G. morhua* (genus *Gadus*)	Pepe et al. [113]
		AFLP	- (genomic DNA)	nDNA	*M. capensis* +*others species from different genus*	Maldini, Marzano, Fortes, Papa and Gandolfi [88]
		PCR-RFLP	*COI*	mtDNA	*M. capensis*, *M. paradoxus*, *M. polli*	Pappalardo and Ferrito [114]
		Real-time PCR, FINS	mtDNA control region (PCR), *cytb* (FINS)	mtDNA	*M. merluccius*	Sánchez et al. [115]
		PCR	*PanI*	nDNA	*Merluccius* spp. *+gadoid species*	Hubalkova, Kralik, Kasalova and Rencova [33]
Pleuronectidae	Flatfish (*Glyptocephalus*, *Hippoglossoides*, *Kareius*, *Lepidopsetta*, *Limanda*, *Microstomus*, *Platichthys*, *Pleuronectes Reinhardtius*, *Verasper*)	FINS	*COI*	mtDNA	more than 50 flatfish species; including also samples from Bothidae, Citharidae, Cynoglossidae, Paralichthyidae, Psettodidae, Rhombosoleidae, Scophthalmidae, and Soleidae family	Espineira et al. [116]
Salmonidae	Salmon (*Salmo*)	LAMP, PCR	*cytb* (LAMP), *COI* (PCR)	mtDNA	*S. salar (in qPCR, other tested species provided Ct app. 30–34)*	Xiong et al. [117]
		real-time LAMP, PCR	*cytb*	mtDNA	*S. salar*	Xiong et al. [118]
		real-time LAMP	*cytb*	mtDNA	*S. salar*	Li, Cheng, Xu, Cui, Cao, Xiong, Wang and Xiong [45]
		real-time PCR	*GH*1, *18S rDNA*	nDNA	*S. salar*	Soga et al. [119]
		SNPs	94 SNPs loci	genome	*S. salar*	Holman et al. [120]
		SNPs	39 SNPs loci	genome	*S. trutta*	Drywa et al. [121]
	Salmon (*Salmo*, *Oncorhynchus*)	real-time PCR	*pvb*	nDNA	*S. salar*, *O.nerka*	Hildebrandt and Garber [122]
		real-time PCR	*ITS*1	nDNA	*S. salar*, *S. trutta* (*O. mykiss, O. gorbuscha and O. keta was amplified with higher Ct value; cut off for this species is Ct* > 27)	Herrero et al. [123]
		AFLP-SCAR	*cytb*	mtDNA	*S. salar*, *O.mykkis*	Zhang and Cai [124]
		PCR-RFLP	*tRNA^Glu^/cytb*	mtDNA	*O. gorbuscha*, *O. keta*, *O. kisutch*, *S. salar* +20 others species from different genus	Wolf, Burgener, Hübner and Lüthy [105]
	Salmon, trout (*Salmo*)	RAPD	-	genome	*S. salar*, *S. trutta*	Elo et al. [125]
	Salmon, trout (*Salmo*, *Oncorhynchus*)	PCR-RFLP	*COII*	mtDNA	*S. salar*, *O. mykkis*	Carrera et al. [126]
		PCR-RFLP	*p*53	nDNA	*S. salar*, *O. mykkis*	Carrera et al. [127]
		PCR-RFLP	*16S rRNA*	mtDNA	*S. salar*, *O. mykkis*	Carrera et al. [128]
		PCR-RFLP	*cytb*	mtDNA	*S. salar*, *O. mykkis*	Carrera et al. [129]
		PCR-RFLP	*COI*	mtDNA	*O. gorbuscha*, *O. keta*, *O. kisutch*, *O. mykkis*, *O. tshawytscha*, *S. salar*	Mueller et al. [103]
		SNPs	566 SNPs loci	genome	*O. mykkis*, *S.salar*, *S.trutta*	Drywa et al. [130]
		real-time LAMP	*cytb*	mtDNA	*O. mykkis*, *S.salar*	Li et al. [131]
		real-time PCR	*COI*, *cytb*	mtDNA	*O. mykkis*, *S.salar*	Xu et al. [132]
	Salmon, trout, bream (*Salmo*, *Oncorhynchus*, *Brama*)	PCR-RFLP, FINS	cytb	mtDNA	*O. clarki*, *O. mykiss*, *O. tschawytscha*, *O. nerka*, *O. gorbuscha*, *O. kisutch*, *O. masou*, *O. keta*, *S. salar*, *S. trutta*, *Brama* spp	Espiñeira et al. [133]
	Salmon, trout, char (*Salmo*, *Oncorhynchus*, *Salvelinus*)	PCR-SSCP	*cytb, GH, pvb*	mtDNA, nDNA	*S. salar*, *S. trutta*, *S. alpinus*, *S. fontinalis*, *O. mykiss*, *O. kisutch*, *O. nerka*, *O. keta*, *O. gorbuscha*, *O. tschawytscha*	Rehbein [134]
		AFLP	- (genomic DNA)	nDNA	*O. keta*, *O.mykkis*, *S. alpinus*, *S. fontinalis* *+others species from different genus*	Maldini, Marzano, Fortes, Papa and Gandolfi [88]
	Salmonids (*Oncorhynchus*, *Salvelinus*, *Hucho*, *Brachymystax*, *Salmo*, *Coregonus*, *Thymallus*)	real-time PCR	*GH*2	nDNA	31 salmonid species	Li et al. [135]
	Trout (*Oncorhynchus*)	PCR, LAMP	*COI* (PCR), *cytb* (LAMP)	mtDNA	*O. mykkis*	Xiong et al. [136]
		SNPs	95 SNPs loci	nDNA	*O. mykkis*	Liu et al. [137], Liu et al. [138]
Scombridae	Mackerel (*Scomber*)	real-time PCR, FINS	*cytb*	mtDNA	*S. scombrus*	Velasco et al. [139]
		PCR-RFLP	5S rDNA nontranscribed spacer	nDNA	*S. japonicus*, *S. australasicus*, *S. scombrus*	Aranishi [140]
		PCR-RFLP	5S rDNA nontranscribed spacer	nDNA	*S. japonicus*, *S. scombrus*	Aranishi [141]
		real-time PCR	*pvb*	nDNA	*S.scombrus*, *T.trachurus*	Prado, Boix and von Holst [90]
	Bonito (*Sarda*)	microsatellites	5 microsatellite loci in genome		*S. sarda* *(loci previously published for other Scombrid fishes)*	Turan [142]
	Tuna (*Thunnus*)	LAMP	*cytb*	mtDNA	*K. pelamis*	Xiong et al. [143]
		PCR-SSCP	*cytb*	mtDNA	*K. pelamis*, *T. alalunga*, *T. albacares*, *T.thynnus*, *T. obesus*	Rehbein et al. [144]
	Bonito, Tuna (*Sarda*, *Thunnus*)	PCR	*cytb*	mtDNA	*S. sarda*, *T. thynnus*	Lockley and Bardsley [145]
	Mackerel, tuna (*Auxis*, *Euthynnus*, *Katsuwonus*, *Scomber*, *Thunnus*)	FINS	*16S rRNA*	mtDNA	*T. thynnus*, *T. albacares*, *T. obesus*, *T. alalunga*, *T. maccoyii*, *T. tonggol*, *T. orientalis*, *A. thazard*, *A. rochei*, *E. affinis*, *E. alletteratus*, *E. lineatus*, *K. pelamis*, *A. fallai*, *S. orientalis*, *S. australis*, *S. chiliensis*, *S. scombrus*, *S. japonicus*, *S. australasicus*, *S. colias* +Fish species from genus Clupeidae, Engraulidae, Salangidae family	Armani, Tinacci, Xiong, Castigliego, Gianfaldoni and Guidi [92]
-	40 animal genus	PCR-SSCP	*cytb*	mtDNA	23 fish species, 19 other animal species	Weder et al. [146]
	Fish genus	real-time PCR	*16S rRNA*	mtDNA	26 fish species (universal primers for fish detection)	Fernandes et al. [147]

* Gene abbreviations: *COI* = cytochrome-c-oxidase subunit I, *COII* = cytochrome-c-oxidase subunit II, *cytb* = cytochrome *b*, *GH* = growth hormone, *ITS1* = internal transcribed spacer 1, *Pan*I = pantophysin I, *Plagl*2 = pleiomorphic adenoma gene-like 2, *p*53 = nuclear protein p53, *pvb* = parvalbumin, *16S rRNA* = 16S ribosomal RNA, *18S rDNA* = 18S ribosomal DNA, *5S rDNA* = 5S ribosomal DNA.

**Table 2:** Comparison of parameters for selected methods.

Parameters	PCR	DNA Barcoding	FINS	RFLP/PCR-RFLP	SSCP	LAMP
Required amount of DNA for analysis *	Low	Medium	Medium	High/medium	Low	Low
Used length of DNA or DNA fragment	Depend on used format (usually 80–600 bp)	usually app. 600 bp; DNA minibarcoding up to 200 bp	Usually 200–800 bp	depends on the size of diagnostic restriction fragments in nuclear DNA or PCR product	Usually up to 250 bp	Up to 250 bp
Capacity of the machine	Mainly 96 reactions in one run	Mainly 96 reactions in one run	Mainly 96 reactions in one run	depending on the capacity of the gel (usually up to 40 holes)/PCR machine (96 reactions)	Mainly 96 reactions in one run (PCR machine)	Mainly 96 reactions in one run (PCR machine) or according to the capacity of the water bath
Duration of the analysis without DNA extraction	45–120 min	Min 24 h	Min 24 h	Min 6 h Evaluation: quick	Min 24 h	15–60 min
Possibility of product detection/visualization	Fluorimetry, agarose gel	Capillary electrophoresis (sequencing chromatogram)	Capillary electrophoresis (sequencing chromatogram)	Agarose gel (for RFLP follow by detection of labeled probes)	Polyacrylamide gel or capillary electrophoresis	Turbidimetry, colorimetry, fluorimetry, agarose gel
Feasibility for analysis of raw products	Yes, reliable	Yes, reliable	Yes, reliable	Yes, reliable	Yes, reliable	Yes, reliable
Feasibility for analysis of heat-treated fish products	Yes, reliable	Yes, depend on DNA degradation rate; minibarcoding can be used	Yes, depend on DNA degradation rate (more applicable for shorter products)	Yes, depend on DNA degradation rate	Yes, reliable	Yes, reliable
Possibility for species identification from mixed samples	Yes	No	No	Yes	Yes	Yes
Possibility of the multiplex analysis	Yes, reliable	No (for Sanger sequencing); possible by metabarcoding	No (for Sanger sequencing)	No/Yes if the specific restriction site is inside PCR amplicons for each identified species	Yes (fluorescence detection)	Yes (potencial for fluorescence detection)
Operation	Simple	Moderate	Moderate	Moderate	Moderate	Simple
Need for obtained data bioinformatical analysis	No	Yes	Yes	No	No	No
Potential for interlaboratory reproducibility	High	High	High	High	Medium	Medium
Potential for database construction	High (amplicon sequence or length)	High	High	Medium (fingerprint)	Medium	No
Usage	Simple to evaluate. The PCR amplicon or fluorescence curve is or is not there, which is clearly visible from the primary results.	Comparison of obtained sequence with available, updated databases. Primers and reaction conditions are verified and available in databases and/or articles.	Necessity of DNA sequences reference samples for the construction of a phylogenetic tree.	Necessity of reference fingerprint database.	Necessity of reference fingerprint database.	Simple to evaluate. The presence of lamplicons are clearly visible on the gel, by turbidimetry or color change of solution.

* Low: ≤100 ng; medium: 50–500 ng, because sequencing depends on the length of the amplicons; high: ≥1 μg.

## Data Availability

Not applicable.

## Supplementary Materials

The following supporting information can be downloaded at: https://www.mdpi.com/article/10.3390/foods12010228/s1, Table S1: Genome attributes data for the sequenced members of 15 fish orders containing commercially significant species.

## Abbreviations

AFLP	Amplified Fragment Length Polymorphism
COI	Cytochrome-c-oxidase subunit I
*cytb*	Cytochrome b gene
DNA	Deoxyribonucleotide Acid
dPCR	Digital PCR
ELISA	Enzyme-Linked Immuno Sorbent Assay
EMBL	European Molecular Biology Laboratory
EU	European Union
FAO	Food and Agriculture Organization of the United Nations
FINS	Forensically Informative Nucleotide Sequencing
FISH-BOL	The Fish Barcode of Life Initiative
GM	Genetically Modified
GMO	Genetically Modified Organism
HRM	High-Resolution Melting
LAMP	Loop-Mediated Isothermal Amplification
mtDNA	Mitochondrial DNA
NCBI	National Center for Biotechnology Information
NGS	Next Generation Sequencing
NMR	Nuclear Magnetic Resonance
nDNA	Nuclear DNA
PCR	Polymerase Chain Reaction
qPCR	PCR with fluorescence detection in real-time
RAPD	Random Amplified Polymorphic DNA
RFLP	Restriction Fragment Length Polymorphism
SSCP	Single Strand Conformation Polymorphism
xMAP	Multi-Analyte Profiling
WGS	Whole-Genome Sequencing
