# Supplementary material for: Identification of Fish Species and Targeted Genetic Modifications Based on DNA Analysis: State of the Art
Source: Foods. 2023 Jan 3;12(1):228. doi: 10.3390/foods12010228 (PMC9818732; doi:10.3390/foods12010228)
Supplement: Supplementary file 1 [file foods-12-00228-s001.zip › foods-2097801-supplementary.pdf]

Identification of fish species and its targeted genetic modifications based on DNA analysis: state of the art

Čermáková Eliška[a,b], Lencová Simona[a], Mukherjee Subham[b, c], Horká Petra[c], Vobruba Šimon[b], Demnerová Kateřina[a], Zdeňková Kamila[a]

[a]Department of Biochemistry and Microbiology, University of Chemistry and Technology, Prague, Technická 5, 166 28 Prague 6, Czech Republic.

[b]Department of Chemistry, Biochemistry and Food Microbiology, Food Research Institute Prague, Radiová 1285/7, 10231 Prague 10, Czech Republic.

[c]Institute for Environmental Studies, Charles University, Faculty of Science, Benatska 2, 128 01 Prague 2, Czech Republic.

Supplementary Table S1

Supplementary Table S1: Genome attributes data for the sequenced members of 15 fish orders containing commercially significant species. The number of species for which whole genome and mtDNA sequences are available is listed under the name of order.

Order	Family	Species	Year	Chr[a]	Size (Mb)	Source[b]
**Salmoniformes**	**Salmonidae**	*Coregonus sp. 'balchen'*	2020	40	2068	GB
		Coregonus clupeaformis	2021	40	2753	GB
WGS - 15 species		*Hucho hucho*	2018	NA	2487	GB
mtDNA - 78 species		*Salvelinus alpinus*	2018	39	2170	GB
		Salvelinus namaycush	2020	42	2346	GB
		Salvelinus	2020	39	2169	ENA
		Oncorhynchus tshawytscha	2017	34	2425	GB
		Oncorhynchus kisutch	2017	30	2369	GB
		Oncorhynchus nerka	2019	29	1927	GB
		Oncorhynchus mykiss	2014	29	2179	GB
		Oncorhynchus keta	2020	37	1853	GB
		Oncorhynchus gorbuscha	2021	27	2690	GB
		Salmo salar	2011	29	2966	GB
		Salmo trutta	2019	40	2371	GB
		Thymallus thymallus	2019	51	1564	GB
**Scombriformes**	**Scombridae**	*Thunnus thynnus*	2018	NA	648	GB
		Thunnus orientalis	2013	NA	787	GB
WGS - 7 species		*Thunnus albacares*	2018	NA	728	GB
mtDNA - 80 species		*Thunnus maccoyii*	2021	24	782	GB
		Scomber colias	2021	NA	814	GB
		Euthynnus affinis	2021	NA	758	GB
	**Stromateidae**	*Pampus argenteus*	2014	NA	350	GB

**Esociformes**<br><br>WGS - 6 species<br>mtDNA - 9 species	**Esocidae**	*Esox lucius*	2014	25	941	GB
		Esox niger	2021	NA	860	GB
		Esox masquinongy	2021	NA	1138	GB
	**Umbridae**	*Novumbra hubbsi*	2021	NA	756	GB
		Dallia pectoralis	2021	NA	856	GB
		Umbra pygmaea	2021	NA	1950	GB
**Gadiformes**<br><br>WGS - 28 species<br>mtDNA - 21 species	**Gadidae**	*Gadus morhua*	2011	23	670	GB
		Gadiculus argenteus	2018	NA	397	GB
		Trisopterus minutus	2018	NA	335	GB
		Melanogrammus aeglefinus	2018	NA	653	GB
		Merlangius merlangus	2018	NA	424	GB
		Gadus chalcogrammus	2018	NA	449	GB
		Pollachius virens	2018	NA	395	GB
		Arctogadus glacialis	2018	NA	429	GB
		Boreogadus saida	2018	NA	412	GB
	**Phycidae**	*Phycis phycis*	2018	NA	346	GB
		Phycis blennoides	2018	NA	417	GB
	**Lotidae**	*Lota lota*	2018	NA	397	GB
		Brosme brosme	2018	NA	413	GB
		Molva molva	2018	NA	437	GB
	**Macrouridae**	*Malacocephalus occidentalis*	2018	NA	350	GB
		Macrourus berglax	2018	NA	400	GB
		Bathygadus melanobranchus	2018	NA	431	GB
		Trachyrincus scabrus	2018	NA	370	GB
		Coryphaenoides rupestris	2018	NA	829	GB
		Trachyrincus murrayi	2018	NA	452	GB
	**Moridae**	*Laemonema laureysi*	2018	NA	306	GB
		Mora moro	2018	NA	345	GB

	**Muraenolepididae**	*Muraenolepis marmorata*	2018	NA	416	GB
	**Melanonidae**	*Melanonus zugmayeri*	2018	NA	433	GB
	**Merluccidae**	*Merluccius capensis*	2018	NA	414	GB
		Merluccius polli	2018	NA	401	GB
		Merluccius merluccius	2018	NA	401	GB
	**Bregmacerotidae**	*Bregmaceros cantori*	2018	NA	1144	GB
**Cypriniformes**	**Cyprinidae**	*Cirrhinus molitorella*	2019	NA	920	GB
		Cyprinus carpio	2014	50	1713	GB
WGS - 53 species		*Sinocyclocheilus rhinocerous*	2015	NA	1655	GB
mtDNA - 874 species		*Sinocyclocheilus anshuiensis*	2015	NA	1632	GB
		Sinocyclocheilus grahami	2015	NA	1750	GB
		Carassius auratus	2015	59	1820	GB
		Labeo rohita	2019	NA	1484	GB
		Poropuntius huangchuchieni	2019	NA	760	GB
		Labeo catla	2020	NA	1232	GB
		Labeo gonius	2020	NA	738	GB
		Onychostoma macrolepis	2020	25	886	GB
		Oxygymnocypris stewartii	2018	NA	1849	GB
		Carassius gibelio	2021	NA	1931	GB
		Cirrhinus cirrhosus	2021	NA	1153	GB
		Labeo calbasu	2021	NA	1042	GB
		Puntigrus tetrazona	2021	25	731	GB
		Sinocyclocheilus anophthalmus	2021	NA	1914	GB
		Sinocyclocheilus maitianheensis	2021	NA	1680	GB
		Tor tambroides	2022	NA	1235	GB
		Rhodeus ocellatus	2020	NA	851	GigaDB
	**Xenocyprididae**	*Anabarilius grahami*	2018	NA	992	GB
		Culter alburnus	2020	NA	1017	GB
		Ctenopharyngodon idella	2021	24	893	GB

	Hypophthalmichthys nobilis	2021	24	857	GB
	Hypophthalmichthys molitrix	2022	NA	825	GB
	Megalobrama amblycephala	2021	24	1110	GB
	Pseudobrama simoni	2020	NA	941	GigaDB
**Danionidae**	*Danio rerio, a zebrafish*	2005	25	1679	GB
	Danio albolineatus	2020	NA	1463	GB
	Danio choprai	2020	NA	1102	GB
	Danio jaintianensis	2020	NA	1122	GB
	Danio tinwini	2020	NA	1497	GB
	Danio kyathit	2020	25	1708	GB
	Danio aesculapii	2020	25	1381	GB
	Danionella dracula	2018	NA	665	GB
	Danionella translucida	2019	NA	735	GB
**Leuciscidae**	*Leuciscus waleckii*	2016	NA	752	GB
	Leuciscus idus	2022	25	1104	GB
	Pimephales promelas	2014	NA	1219	GB
	Abramis brama	2022	25	1067	GB
	Squalius cephalus	2022	25	1040	GB
	Rutilus rutilus	2022	NA	1096	GB
	Vimba vimba	2022	25	1054	GB
**Nemacheilidae**	*Triplophysa siluroides*	2019	NA	583	GB
	Triplophysa tibetana	2019	25	652	GB
	Triplophysa dalaica	2020	25	607	GB
	Triplophysa bleekeri	2020	NA	628	GigaDB
**Gastromyzontidae**	*Beaufortia kweichowensis*	2021	25	449	GB
**Gobionidae**	*Gobio gobio*	2022	25	1550	GB
	Paracanthobrama guichenoti	2021	25	1088	GB
	Gobiocypris rarus	2021	25	959	GB
**Catostomidae**	*Myxocyprinus asiaticus*	2021	50	2581	GB

	**Paedocyprididae**	*Paedocypris sp. Pulau Singkep*	2021	NA	404	GB
**Clupeiformes**	**Denticipitidae**	*Denticeps clupeoides*	2019	20	567	GB
	**Engraulidae**	*Coilia nasus*	2019	24	812	GB
WGS - 8 species	**Clupeidae**	*Tenualosa ilisha*	2018	NA	815	GB
mtDNA - 97 species		*Clupea harengus*	2015	26	807	GB
		Sardina pilchardus	2018	NA	949	GB
		Alosa alosa	2021	24	855	GB
		Alosa sapidissima	2021	24	904	GB
		Limnothrissa miodon	2021	NA	581	GB
**Cichliformes**	**Cichlidae**	*Oreochromis niloticus*	2011	22	1006	GB
WGS - 570 species						
mtDNA - 93 species		*Another 569 members of the order with published whole genome sequence*				
**Perciformes**	**Percidae**	*Perca fluviatilis*	2018	NA	958	GB
		Etheostoma spectabile	2019	24	855	GB
WGS - 128 species		*Perca flavescens*	2019	24	877	GB
mtDNA - 260 species		*Sander lucioperca*	2019	NA	900	GB
		Sander vitreus	2019	NA	783	GB
		Etheostoma cragini	2020	24	643	GB
		Percina caprodes	2020	NA	1012	GB
	**Channichthyidae**	*Chaenocephalus aceratus*	2018	NA	623	GB
		Chionodraco hamatus	2019	NA	829	GB
		Chionodraco myersi	2019	NA	1120	GB
		Pseudochaenichthys georgianus	2020	24	1026	GB
	**Nototheniidae**	*Notothenia coriiceps*	2014	NA	637	GB
		Dissostichus mawsoni	2020	NA	926	GB
		Trematomus bernacchii	2020	NA	867	GB
		Trematomus loennbergii	2020	NA	1211	GB

**Anarhichadidae**	*Anarrhichthys ocellatus*	2019	NA	613	GB
**Stichaeidae**	*Cebidichthys violaceus*	2019	NA	593	GB
**Bovichtidae**	*Cottoperca gobio*	2018	24	609	GB
**Serranidae**	*Epinephelus lanceolatus*	2019	24	1087	GB
	Epinephelus moara	2019	24	1030	GB
	Hypoplectrus puella	2018	NA	612	GB
	Plectropomus leopardus	2019	24	903	GB
	Epinephelus coioides	2019	NA	1024	GB
	Epinephelus fuscoguttatus	2020	24	1047	GB
	Cromileptes altivelis	2020	24	1013	GB
	Serranus cabrilla	2020	NA	631	GB
	Cephalopholis sonnerati	2021	NA	1044	GB
**Bathydraconidae**	*Gymnodraco acuticeps*	2020	NA	997	GB
	Parachaenichthys charcoti	2017	NA	709	GigaDB
**Harpagiferidae**	*Harpagifer antarcticus*	2020	NA	942	GB
**Cottidae**	*Myoxocephalus scorpius*	2018	NA	520	GB
	Cottus rhenanus	2015	NA	564	GB
	Taurulus bubalis	2021	21	615	GB
**Sebastidae**	*Adelosebastes latens*	2021	NA	832	GB
	Helicolenus avius	2021	NA	344	GB
	Helicolenus hilgendorfi	2021	NA	481	GB
	Hozukius emblemarius	2021	NA	834	GB
	Hozukius guyotensis	2021	NA	836	GB
	Sebastiscus albofasciatus	2021	NA	787	GB
	Sebastiscus tertius	2021	NA	772	GB
	Sebastolobus alascanus	2021	NA	859	GB
	Sebastolobus altivelis	2021	NA	852	GB
	Sebastes aleutianus	2021	NA	890	GB

		Sebastes alutus	2021	NA	794	GB
		Sebastes atrovirens	2021	NA	802	GB
		Sebastes auriculatus	2021	NA	795	GB
		Sebastes aurora	2021	NA	796	GB
		Sebastes babcocki	2021	NA	785	GB
		Sebastes baramenuke	2021	NA	809	GB
		Sebastes borealis	2021	NA	466	GB
		Sebastes carnatus	2021	NA	785	GB
		Sebastes caurinus	2021	NA	780	GB
		Sebastes ciliatus	2021	NA	813	GB
		Sebastes constellatus	2021	NA	801	GB
		Sebastes crameri	2021	NA	802	GB
		Sebastes dallii	2021	NA	849	GB
		Sebastes diaconus	2021	NA	826	GB
		Sebastes diploproa	2021	NA	791	GB
		Sebastes elongatus	2021	NA	783	GB
		Sebastes ensifer	2021	NA	791	GB
		Sebastes entomelas	2021	NA	802	GB
		Sebastes exsul	2021	NA	790	GB
		Sebastes fasciatus	2021	NA	796	GB
		Sebastes flavidus	2021	NA	804	GB
		Sebastes glaucus	2021	NA	781	GB
		Sebastes goodei	2021	NA	766	GB
		Sebastes helvomaculatus	2021	NA	801	GB
		Sebastes hopkinsi	2021	NA	845	GB
		Sebastes hubbsi	2021	NA	794	GB
		Sebastes cheni	2021	NA	781	GB
		Sebastes chlorostictus	2021	NA	805	GB
		Sebastes inermis	2021	NA	779	GB

		Sebastes iracundus	2021	NA	813	GB
		Sebastes itinus	2021	NA	787	GB
		Sebastes jordani	2021	NA	782	GB
		Sebastes joyneri	2021	NA	780	GB
		Sebastes kiyomatsui	2021	NA	783	GB
		Sebastes koreanus	2021	NA	782	GB
		Sebastes levis	2021	NA	780	GB
		Sebastes maliger	2021	NA	795	GB
		Sebastes matsubarae	2021	NA	809	GB
		Sebastes melanops	2021	NA	783	GB
		Sebastes melanostomus	2021	NA	791	GB
		Sebastes mentella	2021	NA	794	GB
		Sebastes miniatus	2021	NA	851	GB
		Sebastes minor	2021	NA	776	GB
		Sebastes moseri	2021	NA	793	GB
		Sebastes mystinus	2021	NA	798	GB
		Sebastes nebulosus	2021	NA	788	GB
		Sebastes nigrocinctus	2013	NA	746	GB
		Sebastes nivosus	2021	NA	775	GB
		Sebastes norvegicus	2018	NA	718	GB
		Sebastes nudus	2021	NA	795	GB
		Sebastes oblongus	2021	NA	776	GB
		Sebastes oculatus	2021	NA	798	GB
		Sebastes pachycephalus	2021	NA	791	GB
		Sebastes paucispinis	2021	NA	773	GB
		Sebastes pinniger	2021	NA	790	GB
		Sebastes polyspinis	2021	NA	800	GB
		Sebastes proriger	2021	NA	777	GB
		Sebastes rastrelliger	2021	NA	789	GB

		Sebastes reedi	2021	NA	805	GB
		Sebastes rosaceus	2021	NA	960	GB
		Sebastes rosenblatti	2021	NA	780	GB
		Sebastes ruberrimus	2021	NA	778	GB
		Sebastes rubrivinctus	2021	NA	787	GB
		Sebastes saxicola	2021	NA	791	GB
		Sebastes scythropus	2021	NA	775	GB
		Sebastes semicinctus	2021	NA	794	GB
		Sebastes serriceps	2021	NA	784	GB
		Sebastes schlegelii	2020	24	848	GB
		Sebastes steindachneri	2021	NA	781	GB
		Sebastes taczanowskii	2021	NA	785	GB
		Sebastes thompsoni	2021	NA	773	GB
		Sebastes trivittatus	2021	NA	786	GB
		Sebastes umbrosus	2020	24	801	GB
		Sebastes variabilis	2021	NA	818	GB
		Sebastes variegatus	2021	NA	789	GB
		Sebastes vulpes	2021	NA	524	GB
		Sebastes wilsoni	2021	NA	807	GB
		Sebastes zacentrus	2021	NA	802	GB
		Sebastes zonatus	2021	NA	764	GB
	**Gasterosteidae**	*Apeltes quadracus*	2022	23	429	GB
		Gasterosteus aculeatus aculeatus	2021	21	472	GB
		Pungitius pungitius	2019	NA	467	GB
		Gasterosteus nipponicus	2020	21	600	GB
	**Pholidae**	*Pholis gunnellus*	2021	24	589	GB
	**Hexagrammidae**	*Ophiodon elongatus*	2021	NA	646	GB
**Anabantiformes**	**Osphronemidae**	*Betta splendens*	2018	21	441	GB
	**Channidae**	*Channa argus*	2019	24	644	GB

WGS - 5 species<br>mtDNA - 27 species		*Channa maculata*	2021	21	619	GB
	**Anabantidae**	*Anabas testudineus*	2018	NA	570	GB
	**Helostomatidae**	*Helostoma temminkii*	2018	NA	600	GB
**Siluriformes**<br><br>WGS - 17 species<br>mtDNA - 174 species	**Ictaluridae**	*Ictalurus punctatus*	2016	29	783	GB
		Ameriurus melas	2020	29	868	GB
		Noturus placidus	2022	NA	896	GB
	**Siluridae**	*Ompok bimaculatus*	2019	NA	718	GB
		Silurus glanis	2020	NA	793	GB
		Silurus meridionalis	2020	29	742	GB
	**Auchenipteridae**	*Ageneiosus marmoratus*	2018	NA	1030	GB
	**Sisoridae**	*Bagarius yarrelli*	2019	NA	570	GB
	**Clariidae**	*Clarias batrachus*	2018	NA	821	GB
		Clarias macrocephalus	2020	NA	883	GB
		Clarias magur	2020	NA	941	GB
	**Pangasiidae**	*Pangasianodon hypophthalmus*	2018	NA	715	GB
		Pangasianodon gigas	2022	30	827	GB
	**Bagridae**	*Tachysurus fulvidraco*	2018	NA	713	GB
		Hemibagrus wyckioides	2021	29	790	GB
	**Callichthyidae**	*Corydoras maculifer*	2021	NA	636	GB
		Corydoras sp. C115	2021	NA	700	GB
**Pleuronectiformes**<br><br>WGS - 17 species<br>mtDNA - 73 species	**Pleuronectidae**	*Pseudopleuronectes yokohamae*	2014	NA	548	GB
		Reinhardtius hippoglossoides	2019	NA	678	GB
		Hippoglossus hippoglossus	2019	24	597	GB
		Hippoglossus stenolepis	2020	24	594	GB
		Verasper variegatus	2020	23	545	GB
		Platichthys stellatus	2021	24	610	GB
	**Cynoglossidae**	*Cynoglossus semilaevis*	2014	22	470	GB
		Paraplagusia blochii	2021	NA	555	GB

	**Paralichthyidae**	*Paralichthys olivaceus*	2016	NA	644	GB
		Pseudorhombus dupliciocellatus	2021	NA	395	GB
	**Scophthalmidae**	*Scophthalmus maximus*	2018	22	525	GB
	**Soleidae**	*Solea senegalensis*	2021	21	604	GB
		Brachirus orientalis	2021	NA	630	GB
	**Pserrodoidei**	*Psettodes erumei*	2021	NA	467	GB
	**Rhombosoleidae**	*Colistium nudipinnis*	2021	NA	428	GB
	**Achiridae**	*Trinectes maculatus*	2021	NA	450	GB
	**Bothidae**	*Chascanopsetta lugubris*	2021	NA	578	GB
**Gobiiformes**	**Gobiidae**	*Lesueurigobius sanzi*	2018	NA	810	GB
		Neogobius melanostomus	2019	NA	1003	GB
WGS - 13 species		*Periophthalmodon schlosseri*	2014	NA	679	GB
mtDNA - 136 species		*Periophthalmus magnuspinnatus*	2014	NA	701	GB
		Scartelaos histophorus	2014	NA	695	GB
		Boleophthalmus pectinirostris	2014	NA	695	GB
		Lythrypnus dalli	2020	NA	830	GB
		Chaenogobius annularis	2020	NA	747	GB
		Mugilogobius chulae	2021	22	1003	GB
		Rhinogobius similis	2021	22	890	GB
		Proterorhinus semilunaris	2022	23	865	GB
		Periophthalmus modestus	2021	NA	854	GB
		Bostrychus sinensis	2021	23	886	GB
**Spariformes**	**Sparidae**	*Sparus aurata*	2018	24	833	GB
		Spondyliosoma cantharus	2018	NA	680	GB
WGS - 5 species		*Pagrus major*	2017	NA	875	GB
mtDNA - 27 species		*Acanthopagrus latus*	2020	24	685	GB
		Diplodus sargus	2020	NA	784	GB
**Anguilliformes**	**Anguillidae**	*Anguilla rostrata*	2016	NA	1413	GB

		Anguilla japonica	2013	NA	967	GB
WGS - 6 species		*Anguilla anguilla*	2014	NA	1019	GB
mtDNA - 67 species		*Anguilla megastoma*	2020	NA	878	GB
		Anguilla marmorata	2020	NA	882	GB
		Anguilla obscura	2020	NA	882	GB
**Istiophoriformes**	**Xiphiidae**	*Xiphias gladius*	2021	24	692	GB
WGS - 2 species	**Istiophoridae**	*Istiophorus platypterus*	2021	24	614	GB
mtDNA - 12 species						

[a]Chr = Number of chromosomes, NA = not available; [b]GB = GenBank (https://www.ncbi.nlm.nih.gov/genbank/), ENA = the European Nucleotide Archive (www.ebi.ac.uk/ena), GigaDB = GigaDB Datasets (http://gigadb.org/)
